# Wnt Signaling Mediates LTP-Dependent Spine Plasticity and AMPAR Localization through Frizzled-7 Receptors

**DOI:** 10.1016/j.celrep.2018.03.119

**Published:** 2018-04-25

**Authors:** Faye McLeod, Alessandro Bossio, Aude Marzo, Lorenza Ciani, Sara Sibilla, Saad Hannan, Gemma A. Wilson, Ernest Palomer, Trevor G. Smart, Alasdair Gibb, Patricia C. Salinas

**Affiliations:** 1 Department of Cell and Developmental Biology, University College London, London WC1E 6BT, UK; 2 Department of Neuroscience, Physiology and Pharmacology, University College London, London WC1E 6BT, UK

**Keywords:** Wnt signaling, synaptic plasticity, spine plasticity, LTP, AMPA receptors, Frizzled-7, Sfrps

## Abstract

The structural and functional plasticity of synapses is critical for learning and memory. Long-term potentiation (LTP) induction promotes spine growth and AMPAR accumulation at excitatory synapses, leading to increased synaptic strength. Glutamate initiates these processes, but the contribution from extracellular modulators is not fully established. Wnts are required for spine formation; however, their impact on activity-mediated spine plasticity and AMPAR localization is unknown. We found that LTP induction rapidly increased synaptic Wnt7a/b protein levels. Acute blockade of endogenous Wnts or loss of postsynaptic Frizzled-7 (Fz7) receptors impaired LTP-mediated synaptic strength, spine growth, and AMPAR localization at synapses. Live imaging of SEP-GluA1 and single-particle tracking revealed that Wnt7a rapidly promoted synaptic AMPAR recruitment and trapping. Wnt7a, through Fz7, induced CaMKII-dependent loss of SynGAP from spines and increased extrasynaptic AMPARs by PKA phosphorylation. We identify a critical role for Wnt-Fz7 signaling in LTP-mediated synaptic accumulation of AMPARs and spine plasticity.

## Highlights

• LTP-mediated spine plasticity and AMPAR trafficking require Wnt-Fz7 signaling
• LTP induction rapidly elevates Wnt7a/b protein at spines
• Wnt7a induces the fast recruitment of synaptic and extrasynaptic AMPARs
• Wnt7a-Fz7 promotes synaptic AMPAR localization via CaMKII, PKA, and ERK cascades

## Graphical Abstract

**Figure:**
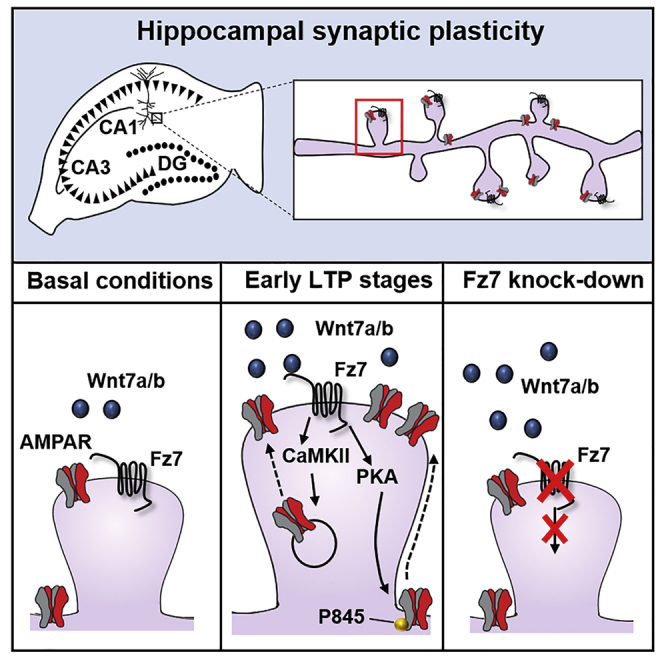

## Teaser

McLeod et al. reveal that Wnt7a-Fz7 signaling is required for LTP-mediated spine plasticity, AMPAR localization, and synaptic strength through the activation of the CaMKII, ERK, and PKA pathways. Their findings demonstrate that extracellular Wnt proteins are crucial upstream initiators of LTP-mediated structural and function synaptic plasticity.

## Introduction

Induction of long-term potentiation (LTP), the cellular correlate of learning and memory, triggers profound changes in the structure and function of excitatory synapses by increasing dendritic spine size and synaptic strength. Significant progress has been made in understanding the intracellular pathways underlying LTP. It is well documented that entry of Ca^2+^ through N-methyl-D-aspartate receptors (NMDARs) and activation of Ca^2+^/calmodulin-dependent protein kinase II (CaMKII) are key signaling events that lead to spine growth and increased numbers of synaptic α-amino-3-hydroxy-5-methyl-4-isoxazole propionic acid receptors (AMPARs) (Herring and Nicoll, 2016, Huganir and Nicoll, 2013, Malenka and Bear, 2004). Both lateral diffusion and exocytosis of AMPARs at the postsynaptic membrane contribute to increased synaptic AMPARs during LTP (Henley and Wilkinson, 2016, Opazo and Choquet, 2011, Penn et al., 2017). Phosphorylation of the AMPAR subunit GluA1 at serine 845 (S845) by protein kinase A (PKA) (Roche et al., 1996) promotes the trafficking of AMPARs to extrasynaptic sites (Esteban et al., 2003, He et al., 2009, Man et al., 2007, Oh et al., 2006, Yang et al., 2008a), which then move to the synapse by lateral diffusion (Borgdorff and Choquet, 2002, Makino and Malinow, 2009). In addition, CaMKII activation leads to membrane insertion and trapping of AMPARs at the synapse during LTP (Esteban et al., 2003, Hayashi et al., 2000, Opazo et al., 2010). Although activation of NMDARs by glutamate initiates these signaling events, the role of other signals in LTP induction is poorly understood.

Synaptic modulators that are regulated by neuronal activity can also contribute to LTP-induced intracellular signaling. For example, brain-derived neurotrophic factor (BDNF), which increases during LTP (Castrén et al., 1993, Verpelli et al., 2010), modulates AMPAR incorporation at the synapse (Caldeira et al., 2007) and promotes spine plasticity (Harward et al., 2016, Tanaka et al., 2008, Zagrebelsky and Korte, 2014). Another major class of synaptic organizers are Wnt-secreted proteins, which are regulated by activity and play a critical role in synapse formation and synaptic transmission (Budnik and Salinas, 2011, Ciani et al., 2015, Dickins and Salinas, 2013). Neuronal activity enhances the release of Wnt proteins at the *Drosophila* neuromuscular junction (NMJ) (Ataman et al., 2008) and increases the expression and/or release of Wnts in hippocampal neurons (Chen et al., 2006, Gogolla et al., 2009, Wayman et al., 2006). Presynaptically, Wnts regulate neurotransmitter release (Cerpa et al., 2008, Ciani et al., 2015). In addition, Wnt proteins postsynaptically increase surface NMDAR levels, promote spine growth, and enhance synaptic strength (Cerpa et al., 2011, Cerpa et al., 2015, Ciani et al., 2011, McQuate et al., 2017). Although a role for Wnts in LTP has been proposed (Cerpa et al., 2011, Chen et al., 2006, Ivanova et al., 2017, Marzo et al., 2016), their precise function in synaptic plasticity and the mechanisms involved remains elusive.

Here we examined the contribution of Wnt signaling to LTP-associated spine plasticity and AMPAR trafficking. Acute blockade of endogenous Wnts in the hippocampus prevented LTP-dependent increase in synaptic strength and AMPAR localization. LTP induction at Schaffer collateral (SC)-CA1 synapses rapidly increased endogenous Wnt7a/b levels in this region of the hippocampus and at dendritic spines. Loss- and gain-of-function studies showed that Frizzled-7 (Fz7) receptors are required for Wnt7a-mediated spine plasticity and AMPAR localization during LTP. Fz7-deficient principal neurons in the hippocampus exhibited an impaired synaptic potentiation following pairing-induced LTP. Moreover, live imaging of surface super-ecliptic pHluorin (SEP)-tagged GluA1 construct (SEP-GluA1) and quantum dot-tagged GluA1 revealed that Wnt7a rapidly increased the number of AMPARs and reduced their mobility at synapses. Similar to LTP induction, Wnt7a through Fz7 increased phosphorylation of GluA1 at S845 and induced both loss of synaptic Ras-guanosine triphosphatase (GTPase)-activating protein (SynGAP) from spines in a CaMKII-dependent manner and activation of the Ras-extracellular signal-regulated kinase (ERK) pathway, a process that contributes to spine growth and AMPAR recruitment at synapses (Araki et al., 2015, Patterson et al., 2010). Collectively, our results identify a role for Wnts, acting postsynaptically through the Fz7 receptor, as key extracellular signals stimulating spine plasticity and AMPAR synaptic localization during the initial stages of LTP.

## Results

### Neuronal Activity Increases Synaptic Wnt7a/b Levels

Previous studies have shown that synaptic activity regulates the expression levels of Wnts and the surface localization of Frizzled (Fz) receptors (Chen et al., 2006, Gogolla et al., 2009, Li et al., 2012, Sahores et al., 2010, Wayman et al., 2006). We therefore examined whether a potentiating stimulus affects the synaptic levels of endogenous Wnt proteins in the hippocampus. We focused on Wnt7a/b for several reasons. First, Wnt7a/b protein is highly expressed in the hippocampus (Ciani et al., 2011). Second, Wnt7a promotes spine growth and synaptic strength (Ciani et al., 2011). Finally, environmental enrichment increases Wnt7a/b levels in the adult hippocampus (Gogolla et al., 2009).

**Figure 1:**
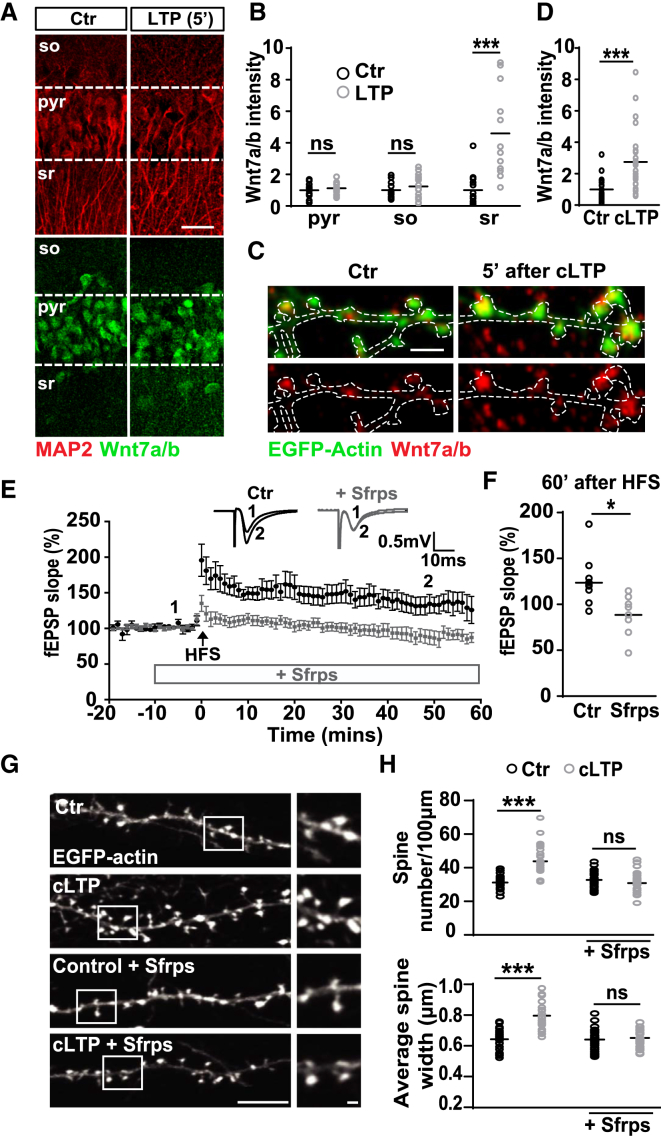
Wnt Proteins Are Upregulated and Required for Structural and Functional Plasticity during LTP (A) Endogenous Wnt7a/b protein (green) in the CA1 pyramidal cell layer (pyr), stratum oriens (so), and stratum radiatum (sr) regions from acute hippocampal slices in control (Ctr) and 5 min after LTP induction. MAP2 (red) used as a reference marker (scale bar: 25 μm). (B) Wnt7a/b fluorescence intensity in control and LTP-induced slices normalized to control levels. n = 11–15 slices from 3 animals (^∗∗∗^p < 0.01, Student’s t test). (C) EGFP-actin-expressing cultured neurons (14 DIV) exposed to control or cLTP conditions. Endogenous Wnt7a/b protein in red (scale bar: 2.5 μm). (D) Wnt7a/b fluorescence intensity (normalized to control) in spines measured 5 min after cLTP treatment. n = 24 cells per condition (^∗∗∗^p < 0.001, Student’s t test). (E) Impact on LTP elicited by HFS in control (black) or Sfrp-treated acute hippocampal slices (gray). Insets show averaged fEPSP recordings before HFS (1) and 50–60 min after HFS (2). Data expressed as mean ± SEM. (F) fEPSPs 60 min after HFS (average of last 10 min of recording) in control and Sfrp-treated slices. n = 8–9 slices from 3–4 animals for each group (^∗^p < 0.05, Student’s t test). (G) Different EGFP-actin-expressing cultured neurons (14 DIV) exposed to control (Ctr) or cLTP conditions in the presence or absence of Sfrps (scale bars: 10 and 1 μm on zoomed image). (H) Spine number (above) and spine width (below). n = 35–40 cells per condition (^∗∗∗^p < 0.001, ANOVA). See also Figures S1 and S2.

Five minutes after high-frequency stimulation (HFS) of the SC fibers in acute hippocampal slices, we observed a significant increase in the levels of Wnt7a/b in the stratum radiatum, where SCs synapse with CA1 cell dendrites (Figures 1A and 1B). This effect was specific to the stimulated area, because no significant changes in Wnt7a/b proteins levels were detected in the CA1 pyramidal cell body layer, stratum oriens, or neighboring cortical regions (layer 4/5) (Figures 1A and 1B; Figures S1A–S1C). In cultured hippocampal neurons, endogenous Wnt7a/b protein was present at dendritic spines and at low levels along the dendritic shaft (Figure 1C). In contrast, following NMDAR-mediated chemical LTP (cLTP) (Lu et al., 2001), endogenous Wnt7a/b levels were elevated at dendritic spines within a comparable time frame to HFS in brain slices (Figures 1C and 1D).

Given the postsynaptic role of Wnt5a in hippocampal neurons (Cerpa et al., 2011, Cerpa et al., 2015), we also examined whether endogenous Wnt5a levels were elevated after induction of cLTP. In contrast to Wnt7a/b, Wnt5a was not elevated at dendritic spines of hippocampal neurons after cLTP (Figures S1D and S1E). Thus, LTP induction specifically increases the levels of endogenous Wnt7a/b protein at excitatory synapses, but not Wnt5a.

### Wnt Proteins Are Required for Functional and Structural Plasticity during LTP

The rapid increase in Wnt7a/b levels following LTP induction suggests that these proteins could contribute to LTP-mediated structural and functional plasticity. To test this, we accutely blocked endogenous Wnts during LTP at the SC-CA1 synapse in acute hippocampal slices using the secreted frizzled-related proteins (Sfrps), which bind and inhibit many endogenous Wnts (Gogolla et al., 2009, Hall et al., 2000, Rosso et al., 2005, Sahores et al., 2010). In controls, HFS produced a robust enhancement in field excitatory postsynaptic potential (fEPSP) slope (34% compared to baseline), which was maintained for at least 1 hr. In contrast, Sfrps abolished LTP induction and maintenance (Figures 1E and 1F; Figure S2A). This effect was not due to changes in basal synaptic transmission, because acute application of Sfrps (15 min) did not affect the NMDAR-to-AMPAR current ratio or input-output measurements (Figures S2B–S2D). Collectively, these findings suggest that endogenous Wnts are required for the induction of LTP.

It is well documented that a long-term increase in synapse strength is correlated with profound structural changes at dendritic spines (De Roo et al., 2008, Kasai et al., 2010). As Wnt7a regulates spine morphogenesis and synaptic strength (Ciani et al., 2011), we investigated the impact of Wnt blockade on LTP-induced spine formation and growth. In control cultured neurons, cLTP induced a significant increase in spine number and size (Figures 1G and 1H) (Fortin et al., 2010, Stamatakou et al., 2013). In contrast, the presence of Sfrps prevented structural changes at dendritic spines following LTP induction (Figures 1G and 1H), suggesting that endogenous Wnts are required for activity-mediated structural plasticity.

### Wnt Antagonists Block LTP-Mediated AMPAR Localization at Innervated Spines

**Figure 2:**
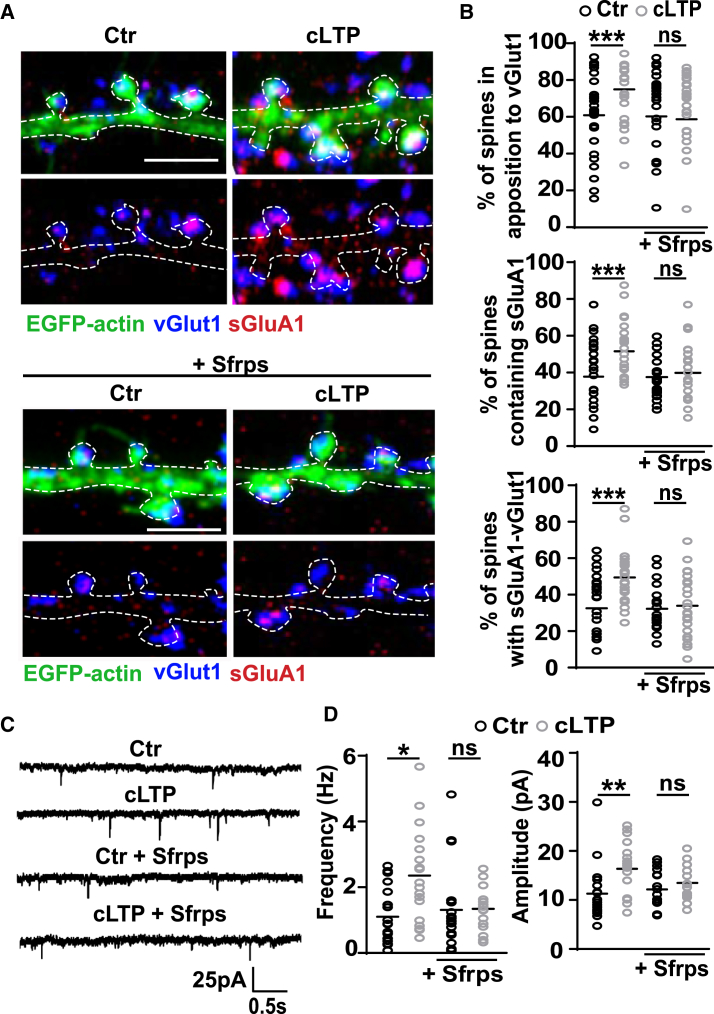
Wnt Blockade Suppresses Activity-Mediated Synaptic Localization of AMPARs (A) Cultured neurons (14 DIV) exposed to control (Ctr) or cLTP conditions, with or without Sfrps. Excitatory presynaptic marker vGlut1 (blue), surface excitatory postsynaptic marker sGluA1 (red), and EGFP-actin (green) (scale bars: 2.5 μm). (B) Percentage of spines containing sGluA1 and in apposition to vGlut1. Percentage of synapses was determined by the colocalization of vGlut1 and sGluA1. n = 39–52 cells per condition (^∗∗∗^p < 0.001, ANOVA). (C) AMPAR-mediated mEPSCs recorded from hippocampal neurons (14 DIV) exposed to control (Ctr) or cLTP conditions in the presence or absence of Sfrps. (D) mEPSC frequency and amplitude. n = 15–19 cells recorded in each group from 6 independent experiments (^∗^p < 0.05 and ^∗∗^p < 0.01, ANOVA). See also Figure S2.

LTP-mediated increase in spine size correlates with enhanced synaptic AMPAR numbers (Kasai et al., 2010, Matsuzaki et al., 2004), contributing to increased synaptic strength (Makino and Malinow, 2009, Shi et al., 1999, Turrigiano et al., 1998). Therefore, we examined whether Wnts affect the surface localization of endogenous AMPARs (sGluA1 and sGluA2) and the innervation of spines containing AMPARs following cLTP induction in cultured hippocampal neurons. cLTP increased the percentage of innervated spines, identified by their colocalization with a presynaptic marker, vesicular glutamate transporter 1 (vGlut1) (Figures 2A and 2B). cLTP also increased the percentage of spines containing AMPAR subunits GluA1 (Figures 2A and 2B) and GluA2 (Figure S2E). The number of AMPAR-containing synapses increased as determined by the colocalization of vGlut1 and sGluA1 (Figure 2B). In contrast, in the presence of Sfrps, cLTP failed to increase the levels of vGlut1, sGluA1, and sGluA2 at dendritic spines (Figures 2A and 2B; Figure S2E). These results demonstrate that Wnt signaling is required for LTP-dependent spine innervation and enhanced AMPAR localization at dendritic spines. Consistently, cLTP increased the frequency and the amplitude of AMPAR-mediated miniature excitatory postsynaptic currents (mEPSCs) (Figures 2C and 2D) as previously reported for this cLTP protocol (Fortin et al., 2010, Lu et al., 2001, Stamatakou et al., 2013). In contrast, Sfrps blocked the increase in both frequency and amplitude of mEPSCs induced by cLTP (Figures 2C and 2D), suggesting that endogenous Wnts facilitate synaptic potentiation and AMPAR mobilization.

### Wnt7a Regulates Synaptic AMPAR Localization

Our findings show that endogenous Wnts are required for activity-mediated plasticity and that Wnt7a/b, both of which increase spine number and size (data not shown), are elevated by LTP. Given the high level of homology between Wnt7a and Wnt7b, we focused our subsequent studies on Wnt7a only. In cultured hippocampal neurons, Wnt7a increased spine innervation (spines colocalized with vGlut1) and spines containing GluA1 and GluA2 AMPAR subunits within 3 hr (Figure S3). Consistently, Wnt7a induced a 2-fold increase in the intensity of sGluA1 puncta at spines (Figure S3B). Concomitant with these changes, the number of synapses containing AMPARs increased, as determined by the colocalization of vGlut1 with sGluA1 (Figure S3). Thus, Wnt7a promotes spine innervation and the synaptic localization of AMPARs.

### Fz7 Receptors Are Required for Wnt7a-Mediated Spine Plasticity and AMPAR Localization during LTP

We next investigated the receptor required for Wnt7a-mediated and activity-dependent spine plasticity and AMPAR localization. Our previous studies showed that Frizzled-5 (Fz5), a Wnt receptor present at synapses, is required for Wnt7a-mediated presynaptic assembly and is regulated by neuronal activity (Sahores et al., 2010). We therefore investigated whether this receptor also functions postsynaptically. However, Fz5 receptors were not enriched at spines in cultured hippocampal neurons (Figure S4A), and gain and loss of function of Fz5 did not affect spine size or number (Figures S4B–S4D). These data suggest that Fz5 does not mediate the effect of Wnt7a on the postsynaptic side.

With the aim to identify other Wnt7a receptors, we performed a binding assay (Sahores et al., 2010). HEK293 cells expressing the Fz receptor cysteine-rich domains (CRDs), which mediate Wnt binding, were exposed to Wnt7a-hemagglutinin (HA). We found that Wnt7a was bound to cells expressing Fz7-CRD (Figure S5A). Furthermore, endogenous Fz7 receptors were enriched in synaptosome fractions (Figure S5B) from brain lysate and were localized to dendritic spines (in contrast, Fz5 did not localize to these postsynaptic structures) in cultured hippocampal neurons (Figure S5C). These studies identify Fz7 as a potential receptor for Wnt7a on the postsynaptic site.

**Figure 3:**
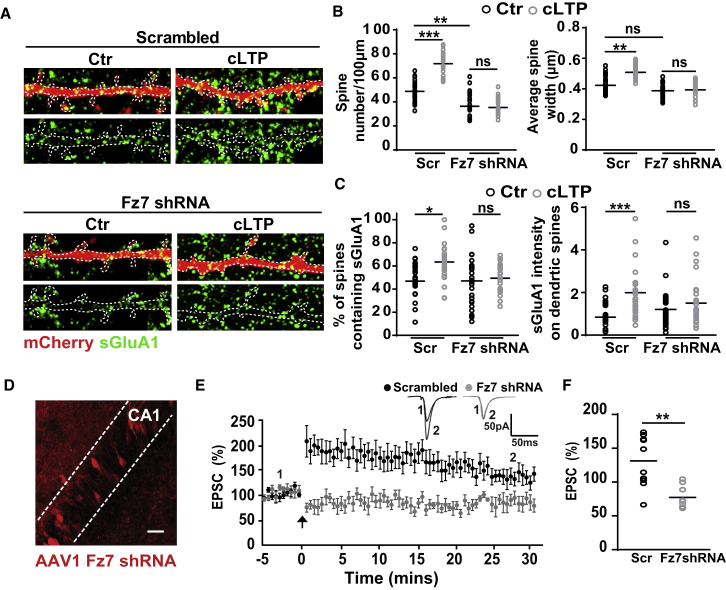
Fz7 Receptors Are Required for Structural and Functional Plasticity and AMPAR Localization during LTP (A) Scrambled or Fz7 shRNA-transfected cultured neurons (14–16 DIV) exposed to control (Ctr) or cLTP conditions. sGluA1 (green) with mCherry-labeled spines (scale bars: 2.5 μm). (B) Spine number and width in neurons exposed to different conditions. n = 25–27 cells per condition (^∗∗^p < 0.01 and ^∗∗∗^p < 0.001, ANOVA). (C) Quantification of surface GluA1 levels at dendritic spines. n = 25–27 cells per condition (^∗^p < 0.05 and ^∗∗∗^p < 0.001, ANOVA). (D) Example image of an organotypic hippocampal slice infected with AAV1 driving the expression of Fz7 shRNA in the CA1 region (scale bar: 25 μm). (E) Sample traces (insets) and summary LTP graph from whole-cell patched neurons in organotypic slices infected with AAV-expressing scrambled or Fz7 shRNA following a pairing protocol (3 Hz stimulation during depolarization to 0 mV; arrow). Data expressed as mean ± SEM. (F) Percentage of EPSC response 30 min after the pairing protocol (average of last 10 min of recording) in scrambled (Scr) and Fz7 shRNA-infected organotypic slices. n = 8–10 cultured slices for each group from four independent cultures (^∗∗∗^p < 0.001, Student’s t test). See also Figures S3–S5 and S7.

To determine whether Fz7 mediates Wnt7a function during synaptic plasticity, we performed loss-of-function studies. Three short hairpin RNA (shRNA) clones against Fz7 were validated in normal rat kidney epithelial (NRK) cells and in cultured hippocampal neurons. qPCR and western blot analyses revealed a significant reduction (60%) in the levels of endogenous Fz7 protein (Figure S5D). Fz7 knockdown reduced the number of dendritic spines (Figures 3A and 3B; Figure S5E), and this defect was rescued by expression of shRNA-insensitive Fz7 cDNA (Figure S5E). Fz7 knockdown had no effect on presynaptic assembly (Figure S5F) but blocked the ability of Wnt7a to increase spine number and size (Figures S5G and S5H). Thus, Wnt7a requires Fz7 receptors on the postsynaptic side to regulate spine plasticity.

Next, we examined the requirement of Fz7 in LTP-dependent spine plasticity and AMPAR localization. cLTP induction failed to promote spine plasticity in Fz7 knockdown neurons compared to scrambled shRNA-expressing neurons (Figures 3A and 3B). cLTP induction did not increase the levels of sGluA1 at dendritic spines in Fz7 knockdown neurons (Figures 3A–3C). We next investigated the impact of Fz7 loss of function in the intact hippocampal circuit. We found that CA1 neurons from organotypic hippocampal cultures (Figure 3D) infected with adeno-associated virus serotype 1 (AAV1)-expressing scrambled shRNA exhibited significant potentiation following a whole-cell pairing protocol as previously described (Barria and Malinow, 2005, Rumpel et al., 2005). In contrast, CA1 neurons expressing Fz7 shRNA did not exhibit potentiation (Figures 3E and 3F). Collectively, these results strongly suggest that Wnt7a-Fz7 signaling is required for hippocampal structural plasticity and AMPAR recruitment to spines during LTP induction.

### Wnt7a Rapidly Increases Spine Size and Postsynaptic AMPAR Localization

**Figure 4:**
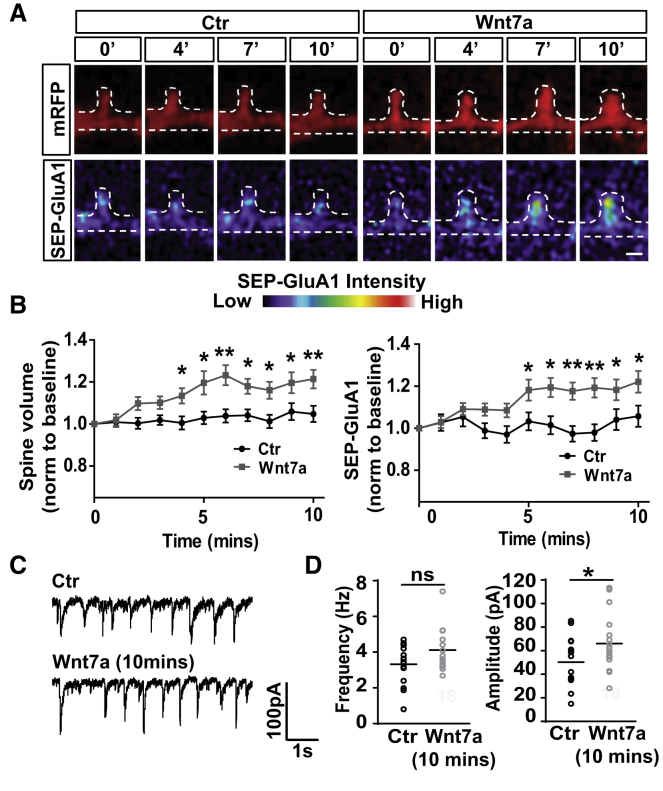
Wnt7a Rapidly Increases AMPAR Localization on Spines and AMPAR Currents (A) mRFP and SEP-GluA1 (pseudo-color) signals in a spine (14 DIV) exposed to control (Ctr) or Wnt7a and imaged over time (scale bar: 1 μm). (B) mRFP volume and SEP-GluA1 intensity at different time points, normalized to baseline. n = 27–33 spines from 7–8 cells (^∗^p < 0.05 and ^∗∗^p < 0.01, ANOVA with repeated measures). Data expressed as mean ± SEM. (C) Spontaneous EPSCs from hippocampal neurons (13–14 DIV) treated with or without Wnt7a for 10 min. (D) Frequency and amplitude of spontaneous EPSC events. n = 18 cells recorded in each group from 5 independent experiments (^∗^p < 0.05, Student’s t test for EPSC amplitude). See also Figure S6.

Spine growth occurs within minutes after LTP followed by the incorporation of GluA1-containing AMPARs into dendritic spines (Kopec et al., 2006, Makino and Malinow, 2009, Tanaka and Hirano, 2012, Yang et al., 2008b). To investigate how fast Wnt7a regulates synaptic localization of AMPARs, we performed live imaging of cultured hippocampal neurons expressing monomeric red fluorescent protein (mRFP) (to delineate neuronal morphology) and SEP-GluA1, which increases in fluorescence upon exposure to a neutral pH in the extracellular environment (Perestenko and Henley, 2006). Wnt7a significantly increased spine size within 4 min, followed closely by elevated SEP-GluA1 levels (Figures 4A and 4B). These results are consistent with our findings that surface levels of endogenous GluA1-containing AMPARs are enriched at dendritic spines 5 min after Wnt7a exposure (Figures S6A and S6B). These effects were specific to Wnt7a, because Wnt5a did not affect endogenous AMPAR localization and spine size (Figures S6C and S6D). Moreover, short-term exposure to Wnt7a did not affect spine number (Figure S6E). In contrast, long-term exposure to Wnt7a (3 hr) affects both spine size and spine number (Figures S5G and S5H) (Ciani et al., 2011). Thus, Wnt7a rapidly increases spine size and GluA1 insertion on dendritic spines comparable to early LTP stages.

Given the rapid effect of Wnt7a on spine growth and AMPAR accumulation, we next investigated the acute effect of Wnt7a on network-driven synaptic strength by examining the amplitude and frequency of spontaneous excitatory postsynaptic currents (sEPSCs) in cultured hippocampal neurons. The frequency of sEPSCs was unaffected (Figures 4C and 4D), consistent with no change in spine number after short-term exposure to Wnt7a (Figure S6E). However, a significant increase in the amplitude of sEPSCs was observed (Figure 4D). These results demonstrate that Wnt7a rapidly enhances synaptic AMPAR localization and synaptic transmission.

### Wnt7a Promotes Immobilization of AMPARs at Spines

**Figure 5:**
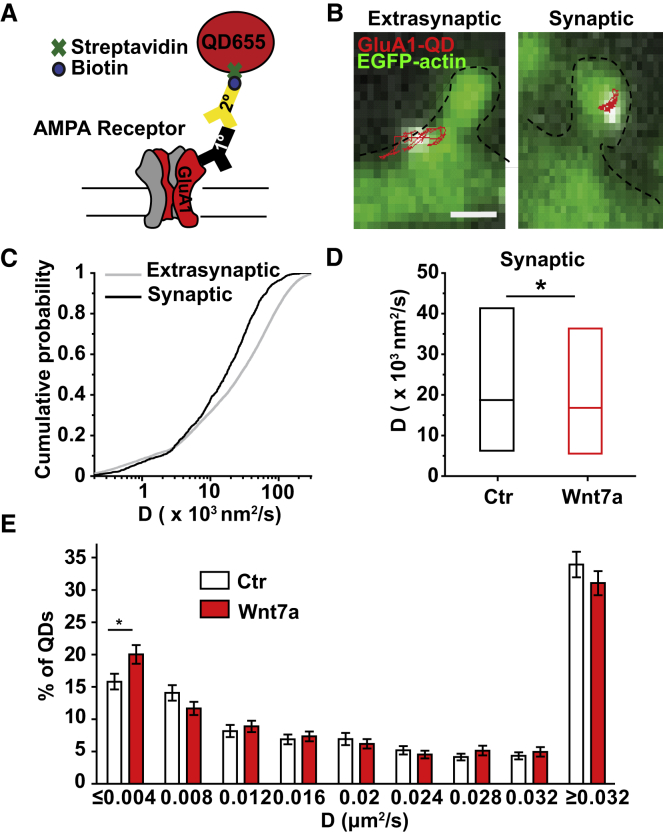
Wnt7a Promotes the Diffusional Trapping of AMPARs in Dendritic Spines (A) Diagram of quantum dot-tagged GluA1 (QD-GluA1). Streptavidin-coupled quantum dots (QDs) recognize biotinylated secondary antibodies bound to GluA1-targeted primary antibodies. (B) Extrasynaptic (left panel) and synaptic (right panel) QD-GluA1 trajectories along the dendrites of hippocampal neurons (13–14 DIV). Spines are visualized with EGFP-actin (scale bar: 1 μm). (C) Cumulative probability of diffusion coefficient (D coefficient) in a log scale between synaptic and extrasynaptic QD-GluA1 in control conditions. (D) Median D coefficient at the synapse in control (Ctr) and Wnt7a-treated cells (10 min) (^∗^p < 0.05, Kolmogorov-Smirnov test). (E) Overall distribution of the D coefficient data shows significant change in the immobile fraction between control (Ctr) and Wnt7a (^∗^p < 0.05, Student’s t test. Data expressed as mean ± SEM. Entire dataset n = 1,638 (control) and 1,409 (Wnt7a) of synaptic trajectories and 12,350 (control) and 10,459 (Wnt7a) of extrasynaptic trajectories from 4 independent experiments. See also Figure S6.

Single-particle tracking techniques have demonstrated that AMPARs are highly mobile on the extrasynaptic plasma membrane and become relatively immobile at the synapse (Borgdorff and Choquet, 2002, Opazo et al., 2010, Petrini et al., 2009). LTP alters the mobility of AMPARs by increasing their lateral diffusion from extrasynaptic sites to the synapse, where they become trapped and less mobile (Makino and Malinow, 2009, Opazo et al., 2010). Because Wnt7a rapidly induced the incorporation of AMPARs into spines (Figure 4), we examined whether Wnt7a affected the lateral movement of GluA1-containing receptors in cultured neurons using time-lapse fluorescence microscopy of quantum dot-tagged GluA1 (QD-GluA1) (Figure 5A). The mobility of synaptic QD-GluA1 was slower than that of extrasynaptic QD-GluA1, defined by the diffusion coefficient (D coefficient, a measure of how far and fast the quantum dots [QDs] are moving) in control neurons (Figures 5B and 5C). At excitatory synapses, these receptors were more confined (indicated by the smaller cell surface area explored in the mean squared displacement [MSD] plot) compared to those located extrasynaptically (Figure S6F). Exposure to Wnt7a for 10 min reduced the diffusion of synaptic QD-GluA1 further in control cells (Figure 5D; Figure S6G), which could reflect a decrease in the mobility of existing receptors or an increased incorporation of receptors into the immobile receptor pool at synapses. The distribution of D coefficients showed that Wnt7a increased the fraction of immobile synaptic QD-GluA1 (Figure 5E). A trend toward increased confinement of synaptic QD-GluA1 in response to Wnt7a was observed (Figure S6F), consistent with our finding that Wnt7a increased the levels of GluA1 at dendritic spines (Figures 4A and 4B; Figures S6A and S6B). Furthermore, Wnt7a increased the diffusion of extrasynaptic QD-GluA1 (velocity of 29,000 nm^2^/s compared to 27,000 nm^2^/s in control cells) (p < 0.001, Kolmogorov-Smirnov test). Altogether, these results suggest that Wnt7a rapidly promotes the recruitment and immobilization of GluA1-containing AMPARs at dendritic spines while increasing the mobility of extrasynaptic AMPARs.

### Wnt7a-Fz7 Signaling Promotes the Extrasynaptic Trafficking of AMPARs through PKA Activation

**Figure 6:**
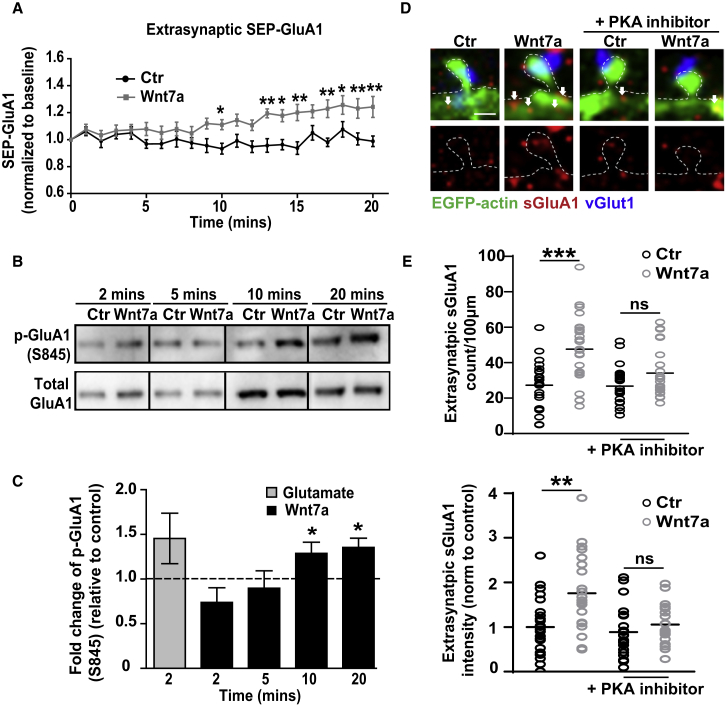
Wnt7a Signals through PKA to Promote Extrasynaptic Localization of AMPARs (A) Extrasynaptic SEP-GluA1 intensity over time in neurons treated with control (Ctr) or Wnt7a, normalized to baseline values. n = 17 dendritic ROIs from 5 cells (^∗^p < 0.05 and ^∗∗^p < 0.01, ANOVA with repeated measures). (B) Phosphorylation of GluA1 at S845 (p-GluA1) following 2–20 min of Wnt7a treatment (14 DIV). (C) p-GluA1 levels normalized to total GluA1 levels. Graphs show fold changes in S845 levels relative to controls (dashed line) over time. Glutamate (50 μM) was used as a positive control. n = 4 experiments per dataset (^∗^p < 0.05, Student’s t test). Data expressed as mean ± SEM. (D) EGFP-actin-expressing hippocampal neurons (14 DIV) exposed to control (Ctr) or Wnt7a for 20 min with or without the PKA inhibitor PKI (14–22) amide. vGlut1 (blue) and sGluA1 (red); arrows indicate extrasynaptic sGluA1 puncta (scale bar: 1 μm). (E) Extrasynaptic sGluA1 puncta normalized to dendritic length, and intensity of extrasynaptic sGluA1 normalized to control. n = 23–27 cells per condition (^∗∗^p < 0.01 and ^∗∗∗^p < 0.001, ANOVA). See also Figure S7.

Phosphorylation of GluA1 at S845 by PKA (Roche et al., 1996) is important for the extrasynaptic surface delivery of AMPARs and LTP expression (Esteban et al., 2003, He et al., 2009, Man et al., 2007, Oh et al., 2006, Yang et al., 2008a). LTP induction increases phosphorylation at this site within 10 min (Oh et al., 2006, Diering et al., 2016). We therefore investigated whether Wnt7a regulates extrasynaptic AMPAR localization. Time-lapse recordings of cultured neurons expressing SEP-GluA1 demonstrated that Wnt7a rapidly increased the levels of extrasynaptic AMPARs (Figure 6A).

Next, we found that Wnt7a progressively increased the phosphorylation of GluA1 at S845, with a maximum effect after 10–20 min (Figures 6B and 6C). Wnt7a-induced phosphorylation of S845 was blocked in hippocampal neurons infected with AAV1 Fz7 shRNA compared to cells infected with control scrambled shRNA (Figures S7A and S7B). In addition, phosphorylation of S845 was required for Wnt7a-mediated extrasynaptic AMPAR recruitment, because inhibition of PKA with myristoylated protein kinase inhibitor (PKI) amide (14–22) significantly blocked the recruitment of extrasynaptic surface AMPARs evoked by Wnt7a (Figures 6D and 6E). Collectively, these findings demonstrate that Wnt7a-Fz7 signaling promotes GluA1 phosphorylation at S845 as observed during early phases of LTP, resulting in increased levels of extrasynaptic AMPARs.

### Wnt7a, through Fz7 and CaMKII Activation, Induces the Loss of SynGAP from Spines and Activation of the Ras-ERK Pathway

**Figure 7:**
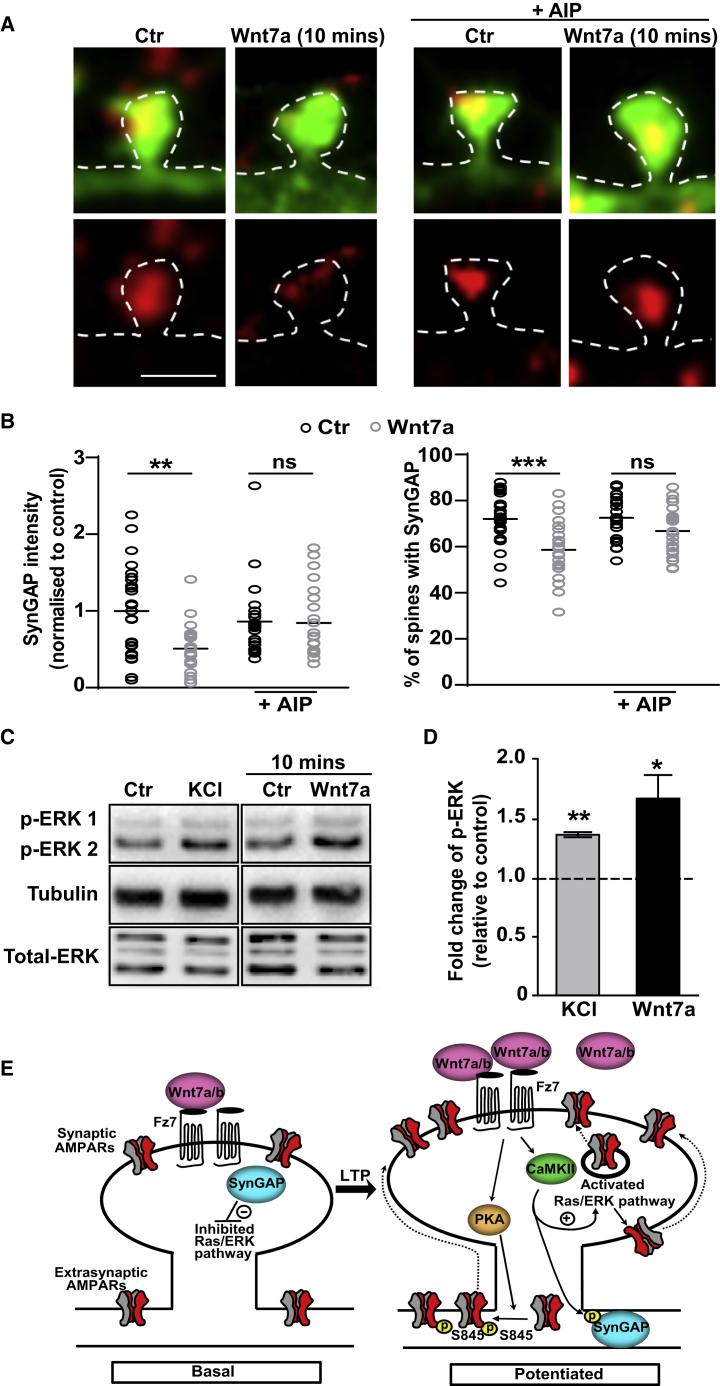
Wnt7a-Mediated Reduction of SynGAP in Spines and Activation of the Ras-ERK Pathway (A) Spines from EGFP-actin-expressing neurons (14 DIV) with endogenous SynGAP (red) exposed to control (Ctr) or Wnt7a for 10 min with or without the CaMKII inhibitor AIP (scale bar: 1 μm). (B) SynGAP intensity within small to medium spines normalized to control levels, and the percentage of spines containing SynGAP. n = 25 cells per condition (^∗∗^p < 0.01 and ^∗∗∗^p < 0.001, ANOVA). (C) Phosphorylated ERK (p-ERK1 and p-ERK2) 10 min after exposure to Wnt7a (14 DIV). Tubulin was used as a loading control. (D) Quantification of p-ERK levels normalized to total ERK levels. Graphs show fold change in p-ERK levels relative to controls (dashed line). Potassium chloride (KCl; 60 mM) was used as a positive control (^∗^p < 0.05 and ^∗∗^p < 0.01, Student’s t test). Data expressed as mean ± SEM. (E) Proposed model for the mechanism by which Wnt7a-Fz7 signaling regulates LTP-induced AMPAR localization and spine growth. LTP induction increases endogenous Wnt7a/b levels at synapses. Wnt7a/b binding to Fz7 activates PKA and CaMKII, resulting in increased levels of extrasynaptic and synaptic AMPARs through the loss of SynGAP at synapses. Dashed arrows represent potential mechanisms. See also Figures S6 and S7.

During LTP, CaMKII activation at dendritic spines promotes the surface insertion and trapping of synaptic AMPARs (Esteban et al., 2003, Hayashi et al., 2000, Opazo et al., 2010). We previously reported that Wnt7a rapidly increases the local activation of CaMKII at dendritic spines and inhibition of CaMKII blocks Wnt7a-mediated increase in spine growth and synaptic strength (Ciani et al., 2011). In agreement with this, Wnt7a promoted the rapid recruitment of AMPARs to dendritic spines through activation of CaMKII (Figure S6H). CaMKII phosphorylates several proteins in the postsynaptic density (PSD) that modulate synaptic AMPAR levels (Huganir and Nicoll, 2013, Lisman et al., 2012). One of these is the SynGAP, which inhibits the Ras-ERK signaling pathway, critical for AMPAR recruitment to spines following LTP induction (Araki et al., 2015, Patterson et al., 2010, Zhu et al., 2002). CaMKII activation leads to the loss of SynGAP from dendritic spines, resulting in increased levels of synaptic AMPARs (Araki et al., 2015). We measured the endogenous levels of SynGAP specifically on small to medium dendritic spines in cultured hippocampal neurons, where Wnt7a rapidly increases CaMKII activity (Ciani et al., 2011). Wnt7a decreases the intensity of SynGAP by ∼50% and the percentage of spines containing SynGAP puncta (Figures 7A and 7B). These events were blocked in cells expressing Fz7 shRNA (Figures S7C and S7D), indicating that Fz7 receptors are required for Wnt7a-induced removal of SynGAP from dendritic spines. Consistent with a role for CaMKII, these effects were prevented by autocamtide – 2 – related inhibitory peptide (AIP), a specific CaMKII inhibitor (Figures 7A and 7B). Furthermore, Wnt7a elevated the levels of phospho-ERK, a marker of ERK pathway activation (Figures 7C and 7D). Altogether, these results demonstrate that Wnt7a-Fz7 signaling induces the loss of SynGAP from dendritic spines in a CaMKII-dependent manner, with the concomitant activation of the Ras-ERK pathway, thereby promoting synaptic AMPAR localization.

## Discussion

Here, we uncovered a role for Wnt-Fz7 signaling in LTP-dependent spine plasticity and synaptic localization of AMPARs. We demonstrated that LTP induction increased Wnt7a/b protein at dendrites and spines. Direct blockade of endogenous Wnts impaired LTP-mediated structural spine plasticity and the synaptic targeting of GluA1-containing AMPARs. We identified Fz7 as the postsynaptic receptor for Wnt7a required for spine plasticity and LTP induction. We also demonstrated that Wnt signaling regulates the membrane localization of AMPARs through PKA-dependent phosphorylation of GluA1 and facilitated the synaptic localization of these receptors by CaMKII-mediated loss of SynGAP at dendritic spines (see model in Figure 7E). Our findings reveal a critical role for Wnts in LTP induction, because these secreted factors trigger downstream signaling events that are required for NMDAR-mediated structural and functional plasticity.

The molecular mechanisms that contribute to the structural and functional plasticity of synapses have been extensively studied. The central role of the neurotransmitter glutamate as the main initiator of LTP-mediated synaptic plasticity is widely recognized; however, the identity and function of secreted molecules that support this role of glutamate is not fully understood. While extracellular signals such as BDNF are recognized as LTP mediators (Zagrebelsky and Korte, 2014), the importance of other extracellular signaling proteins, including Wnts, in LTP-mediated structural and functional plasticity is unclear.

Wnt5a regulates NMDAR-mediated synaptic transmission and LTP expression, but the effect of this Wnt protein is slow, taking approximately 20 min (Cerpa et al., 2011). Similarly, Wnt5a requires 1–2 hr to enhance dendritic spine size and synapse formation in cultured hippocampal neurons and 30 min to regulate spine number (Ramírez et al., 2016). Here, we demonstrate that Wnt5a does not induce fast changes in AMPAR localization or dendritic spine size. Collectively, these findings suggest that Wnt5a may function during later stages of LTP.

Here we discover a unique role for Wnt7a/b at early stages of LTP. HFS of SCs, which induces LTP at CA3-CA1 synapses, specifically increased the levels of endogenous Wnt7a/b protein in the stratum radiatum and is rapidly elevated (5 min) at dendritic spines following cLTP. Given the expression of Wnt7a and Wnt7b at principal neurons in the hippocampus, Wnt7a/b protein may come from presynaptic and/or postsynaptic neurons in the stratum radiatum. Although the mechanisms controlling the rapid accumulation and/or release of Wnt7a/b remain to be elucidated, Wnts could be stored and then released from exosomes at the synapse, as observed at the *Drosophila* NMJ (Budnik et al., 2016, Padamsey et al., 2017). Further studies are required to demonstrate the mechanism by which activity regulates the synaptic accumulation and secretion of Wnts.

Wnt7a, through Fz7 receptors, modulated LTP-associated structural and functional plasticity. Acute blockade of endogenous Wnts with Sfrps suppresses the structural plasticity of dendritic spines and LTP induction and maintenance. This result is consistent with our previous work showing that long-term *in vivo* blockade of endogenous Wnts with Dkk1, another secreted Wnt antagonist, affects LTP (Marzo et al., 2016). Early LTP events depend on the precise regulation of AMPAR content (Henley and Wilkinson, 2016, Huganir and Nicoll, 2013, Temkin et al., 2017). Consistently, Wnt7a rapidly increased the accumulation of AMPARs at dendritic spines, thereby elevating AMPAR-mediated excitatory postsynaptic current (EPSC) amplitude. Using live imaging of SEP-GluA1-expressing neurons and single-particle tracking, we showed that Wnt7a rapidly increased spine size and synaptic AMPAR levels while increasing the number of immobile synaptic AMPARs. The timing of these events is similar to those observed after LTP induction (Kopec et al., 2006, Makino and Malinow, 2009, Tanaka and Hirano, 2012, Yang et al., 2008b). We demonstrated that Wnt7a specifically regulated spine plasticity and AMPAR localization through Fz7 receptors, which were located postsynaptically. Fz7 loss of function prevented cLTP-induced AMPAR accumulation at the surface and inhibits synaptic potentiation following a whole-cell LTP pairing protocol. Thus, our studies delineate a pathway modulating structural and functional plasticity through the Wnt7a-Fz7 signaling cascade.

What is the mechanism by which Wnt7a-Fz7 signaling enhances AMPAR localization and synaptic strength? Following induction of LTP, the number of AMPARs rapidly increases at extrasynaptic sites (Makino and Malinow, 2009, Yang et al., 2008a) through PKA-dependent phosphorylation of GluA1 at S845 (He et al., 2009, Man et al., 2007, Oh et al., 2006, Yang et al., 2008a). Although this phosphorylation site is not required for direct synaptic AMPAR incorporation (Esteban et al., 2003), extrasynaptic receptors can be recruited to the synapse through lateral diffusion in the surface membrane (Bassani et al., 2013, Opazo and Choquet, 2011). A study showed that the rapid recruitment of AMPARs to synapses from an extrasynaptic pool through lateral diffusion and activity-dependent trapping of these receptors at the PSD is imperative for LTP induction (Penn et al., 2017). Moreover, the replenishment of the extracellular pool of AMPARs is through exocytosis and is required to maintain LTP (Penn et al., 2017). Here, we demonstrate that Wnt7a through Fz7 increased PKA-mediated phosphorylation of the GluA1 subunit at S845 and promoted the localization of AMPARs at extrasynaptic sites within 10–20 min. These findings, together with our time-lapse recordings and single-particle tracking data, are consistent with the model proposed by Penn et al. (2017). Thus, our results suggest that Wnts could play an important role in AMPAR synaptic recruitment at early and later stages of LTP.

Activation of CaMKII is also required for the localization and confinement of AMPARs at synaptic sites (Esteban et al., 2003, Herring and Nicoll, 2016, Hayashi et al., 2000, Opazo et al., 2010). Although CaMKII phosphorylates AMPARs at S831, this post-translational modification is not required for AMPAR synaptic localization (Hayashi et al., 2000, Opazo et al., 2010). In contrast, CaMKII phosphorylates SynGAP, a Ras-GTPase that inhibits ERK signaling (Rumbaugh et al., 2006), resulting in the loss of this protein from dendritic spines with the concomitant increase in AMPARs (Araki et al., 2015). Consistent with this model, and in contrast to results obtained with Wnt5a (Codocedo et al., 2015), we found that Wnt7a through Fz7 rapidly promotes the loss of SynGAP from spines in a CaMKII-dependent manner, resulting in the activation of the Ras-ERK pathway. Thus, our findings suggest that Wnt7a-CaMKII signaling regulates the synaptic localization of AMPARs through rapid changes in SynGAP localization.

In summary, our findings reveal a crucial role for Wnt7a-Fz7 signaling in LTP-mediated synaptic plasticity. We demonstrate that endogenous Wnts in the hippocampus are required for LTP induction and localization of AMPARs to dendritic spines by modulating signaling pathways implicated in LTP. Thus, our results identify a mechanism that modulates the initial stages of LTP through Wnt-Fz signaling.

## Experimental Procedures

### Cultures, Cell Transfection, and Constructs

All procedures involving animals were conducted according to the Animals Scientific Procedures Act UK (1986) and in compliance with the ethical standards at University College London (UCL). Rat primary hippocampal cultures were prepared as described previously (Dotti et al., 1988) and maintained for 13–14 days *in vitro* (DIV). Neurons were transfected using calcium phosphate with EGFP-actin, mRFP, or mCherry to visualize dendritic spines or expressed SEP-tagged GluA1 construct (SEP-GluA1) to examine AMPAR trafficking to the cell surface. To investigate the localization and function of Fz (Fz5 and Fz7) receptors, hippocampal cultures were transfected using calcium phosphate with scrambled and shRNA or with full-length receptor. To rescue the phenotype of Fz7 shRNA, hippocampal neurons were transfected with a plasmid containing both Fz7 shRNA and Fz7-insensitive cDNA. Neuronal health was morphologically evaluated before experimental measurements were conducted. Organotypic hippocampal slice cultures were prepared as previously described from postnatal day (P) 6 rats (De Simoni and Yu, 2006). See Supplemental Experimental Procedures for further information.

### Chemical LTP

LTP was induced in cultured hippocampal neurons with a glycine-mediated form of cLTP as previously described (Fortin et al., 2010, Stamatakou et al., 2013). To block Wnts, a combination of recombinant Sfrp1 (2.5 μg/mL; R&D Systems, Abingdon, UK) and Sfrp3 (250 ng/mL; R&D Systems) was used throughout the induction of cLTP and during the incubation with control solution. See Supplemental Experimental Procedures for more information.

### Immunofluorescence, Image Acquisition, and Analyses

Hippocampal neurons were fixed with 4% paraformaldehyde (PFA)/4% sucrose in PBS for 20 min at room temperature, permeabilized with 0.05% Triton, blocked with 5% BSA, and then incubated with primary antibodies overnight at 4°C. For surface AMPAR staining, live cells were incubated with each antibody for 10–15 min at 37°C before fixation. Images were captured on an Olympus FV1000 inverted confocal microscope. For each experiment, 8–12 images of cells were taken per condition and analyzed using Volocity software (Improvision, Llantrisant, UK). Dendritic spine morphology was measured manually. For every EGFP-actin/mCherry-expressing cell, 3–4 dendrites (∼50–100 μm in length each) containing roughly 100 spines were cropped from maximum projections. Spine width was quantified by placing the line tool (in Volocity software) over the maximum spine head width, and the number of spines was counted and normalized to the length of the dendrite. See Supplemental Experimental Procedures for more details.

### Electrophysiology

AMPAR-mediated mEPSCs and sEPSCs were recorded from hippocampal neurons (250 cells/mm^2^) and voltage-clamped at −60 mV in the whole-cell configuration using borosilicate glass patch electrodes. NMDA/AMPAR ratio experiments and input-output experiments were performed in acute transverse hippocampal slices (300 μm) from P28 rats in recording solution. NMDAR EPSCs were recorded at +40 mV, and AMPAR EPSCs were recorded at −40 mV. Recording glass microelectrodes were positioned in the CA1 pyramidal cell layer, while concentric bipolar stimulating electrodes were placed in SC afferent fibers. The peak averaged NMDAR EPSCs were divided by the averaged AMPAR EPSCs. See Supplemental Experimental Procedures for more details.

For fEPSP recordings, glass microelectrodes were positioned in the stratum radiatum of the CA1 region, while concentric bipolar stimulating electrodes were placed in SC afferent fibers to record fEPSPs. Paired pulse stimuli were given 50 ms apart every 10 s, with a stimulation strength set to approximately 50% of the strength giving a maximal response. Following a stable 20-min baseline recording (<10% change in fEPSP slope), LTP was induced by HFS (a single train of 100 stimuli at 100 Hz), and fEPSPs were monitored for 1 hr. To block Wnts, Sfrp3 (250 ng/mL) was applied 10 min before HFS and present during LTP. Pairing LTP experiments were performed in organotypic hippocampal slices (9–16 DIV) infected with AAV1-expressing scrambled or Fz7 shRNA constructs. Slices were continuously perfused at room temperature with oxygenated (95% O_2_/5% CO_2_) recording solution. Synaptic responses were evoked with concentric bipolar stimulating electrodes placed in SC afferent fibers (single-voltage pulses of 200 μs). Pairing LTP was induced in CA1 neurons by pairing 3 Hz presynaptic stimulation of the SCs with 0 mV postsynaptic depolarization. See Supplemental Experimental Procedures for further information.

### Live Imaging of SEP-GluA1 and Analyses

Time-lapse experiments of SEP-GluA1 were performed on an Olympus FV1000 inverted confocal microscope using a 60× oil-immersion objective (numerical aperture [NA] = 1.40) with a temperature-controlled chamber maintained at 37°C in HEPES buffered solution. The z stacks (∼10 frames) were acquired every minute for at least 20 min. Data were analyzed on the projected z stack using ImageJ software from manually selected regions of interest (ROIs). See Supplemental Experimental Procedures for more details.

### Single-Particle Tracking and Quantitative Analyses of Lateral Diffusion

Single-particle tracking and analysis of QD655-tagged AMPARs was performed as previously described (Hannan et al., 2016). See Supplemental Experimental Procedures for details.

### Statistical Analyses

All data are represented as mean in aligned dot plots unless otherwise stated. Data were obtained from 3 independent experiments unless otherwise stated in the figure legends. We assessed our data for normality using Shapiro-Wilk or Kolmogorov-Smirnov tests. Normally distributed data were analyzed using Student’s unpaired or paired (western blots) t test, one-way and two-way ANOVA with Tukey’s post hoc test, or two-way ANOVA with repeated measures. Mann-Whitney, Kruskal-Wallis, and Kolmogorov-Smirnov tests were used for non-parametric data. Statistical significance was accepted as ^∗^ p < 0.05, ^∗∗^ p < 0.01, and ^∗∗∗^ p < 0.001, and non-significant data were indicated as NS.

## Supplemental Information

Document S1. Supplemental Experimental Procedures and Figures S1–S7

Document S2. Article plus Supplemental Information
