# Supplementary material for: Wnt Signaling Mediates LTP-Dependent Spine Plasticity and AMPAR Localization through Frizzled-7 Receptors
Source: Cell Rep. 2018 Apr 25;23(4):1060–71. doi: 10.1016/j.celrep.2018.03.119 (PMC5946458; doi:10.1016/j.celrep.2018.03.119)
Supplement: Document S1. Supplemental Experimental Procedures and Figures S1–S7 [file mmc1.pdf]

Cell Reports, Volume *23*

Supplemental Information

Wnt Signaling Mediates LTP-Dependent

Spine Plasticity and AMPAR Localization

through Frizzled-7 Receptors

Faye McLeod, Alessandro Bossio, Aude Marzo, Lorenza Ciani, Sara Sibilla, Saad Hannan, Gemma A. Wilson, Ernest Palomer, Trevor G. Smart, Alasdair Gibb, and Patricia C. Salinas

SUPPLEMENTAL INFORMATION

SUPPLEMENTAL FIGURES

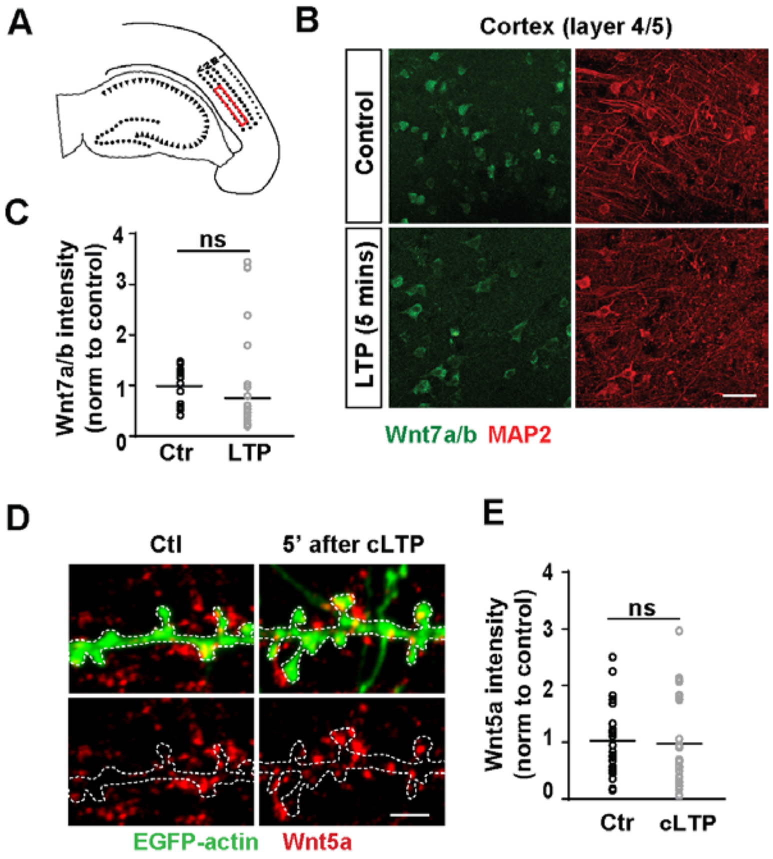

Figure S1: related to Figure 1. Cortical Wnt7a/b or hippocampal Wnt5a levels do not change 5 minutes after synaptic stimulation. (A) Schematic of the cortical area (layers 4/5) imaged for analysis (red box). **(B)** Representative images of endogenous Wnt7a/b staining in control and 5 minutes after LTP induction in the hippocampus. MAP2 (red) used as a reference marker. Scale bar: 25 µm. **(C)** Quantification of cortical Wnt7a/b fluorescence intensity in control and LTP-induced slices normalized to control levels (n = 7 slices from 3 independent experiments, Student's t-test). **(D)** EGFP-actin–expressing cultured hippocampal neurons (12-14 DIV) exposed to control or cLTP conditions. Endogenous Wnt5a protein in red (scale bar: 2.5 µm). **(E)** Quantification of Wnt5a fluorescence intensity (normalized to control) in spines measured 5 minutes after cLTP treatment (n = 25-26 cells per condition, Student's t-test).

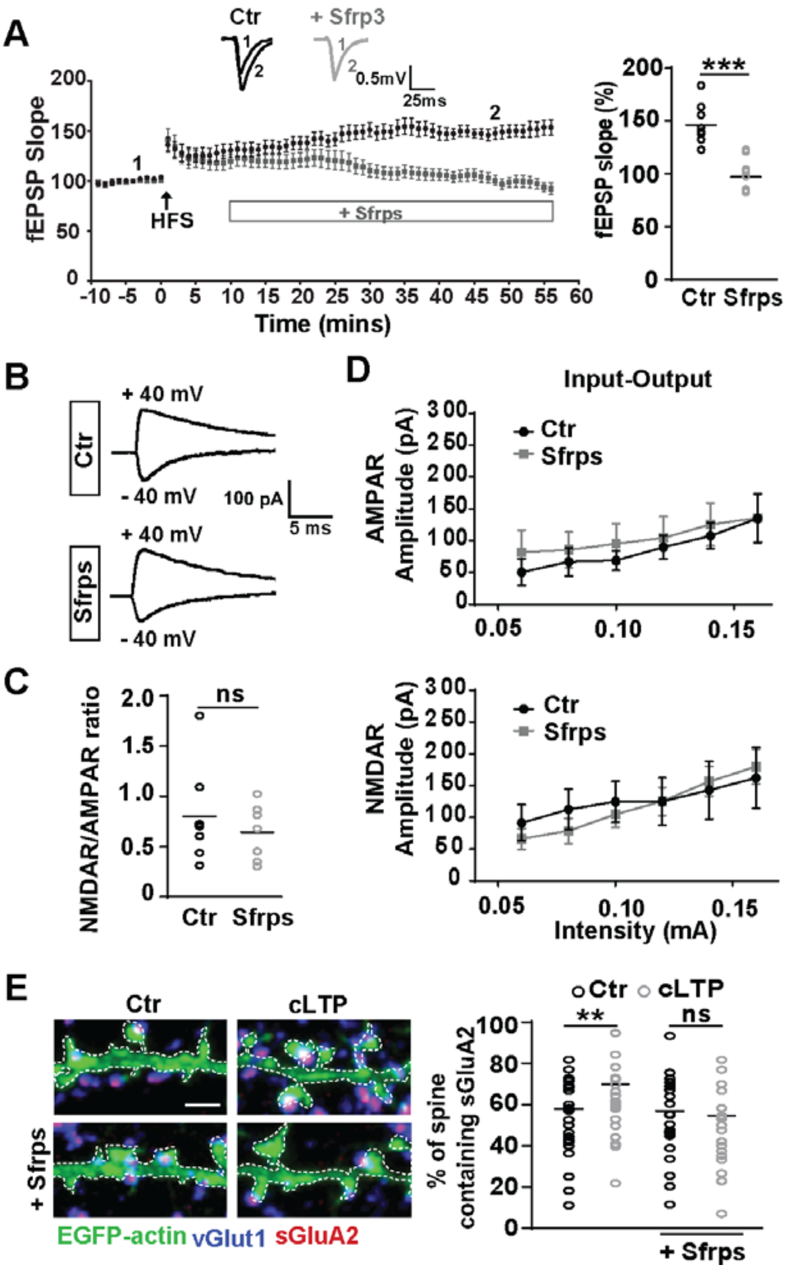

Figure S2: related to Figures 1 and 2. Sfrps impair the maintenance of LTP and activity-dependent sGluA2 AMPAR localization but do not affect short-term basal synaptic transmission. (A) Left: impact on LTP maintenance elicited by HFS in control (in black) or Sfrps treated hippocampal slices (in grey). Insets show representative averaged fEPSP recordings before (1) and after HFS (2). Data expressed as mean ± SEM. Right: quantification of fEPSPs 60 minutes after HFS (average of last 10 minutes of recording) in control and Sfrps treated slices (*n* = 8-9 slices per condition from 6 animals, ***P < 0.01, Student's *t*-test). **(B)** Representative evoked NMDAR (+ 40 mV; in the presence of 10µm CNQX) and AMPAR (- 40mV) EPSC traces from control and Sfrps treated (15 mins) acute hippocampal slices. **(C)** Ratio of NMDAR- to AMPAR-mediated EPSCs (*n* = 7-8 cells for each condition from 4 independent experiments, Student's *t*-test). **(D)** Summary graphs of the input-output measurements for each condition (*n* = 7-8 cells for each condition from 4 independent experiments, ANOVA with repeated measures). Data expressed as mean ± SEM. **(E)** Hippocampal neurons (14 DIV) exposed to control or cLTP conditions, with or without Sfrps. Excitatory presynaptic marker vGlut1 (in blue), surface GluA2 (sGluA2; in red) and EGFP-actin (in green; scale bar: 2.5 µm). Quantification of the percentage of spines containing sGluA2 (n = 39-52 cells per condition, **P < 0.01, ANOVA).

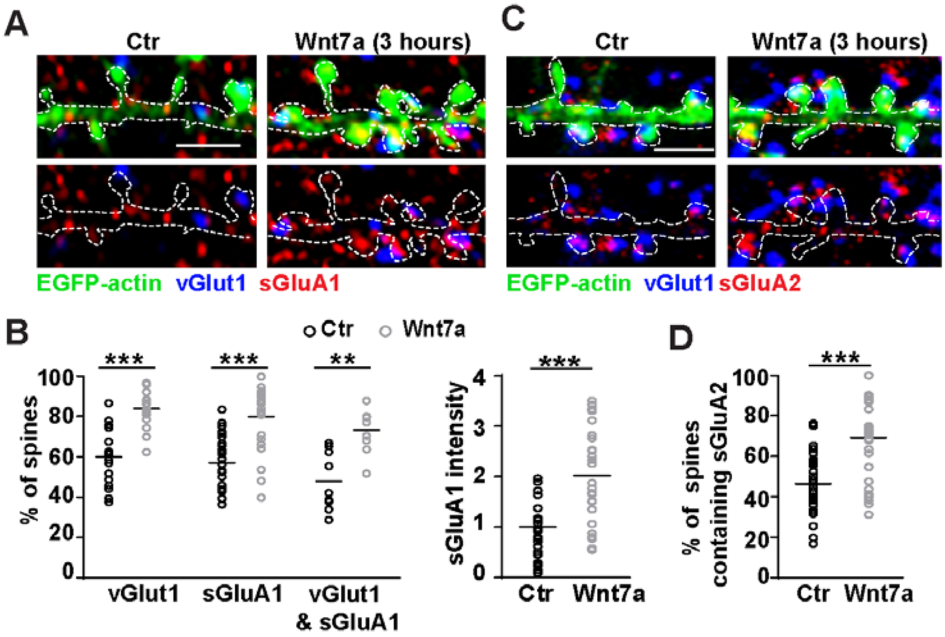

Figure S3: related to Figure 3. Long-term impact of Wnt7a on surface AMPARs. (A) Hippocampal neurons (13-14 DIV) treated with control or Wnt7a for 3 hours. vGlut1 (blue) and sGluA1 (red) on dendritic spines visualized by EGFP-actin (green; scale bar: 2.5 µm). **(B)** Percentage of innervated spines (opposed to vGlut1) and spines containing sGluA1 or synapses (co-localization of vGlut1 and sGluA1). sGluA1 intensity in spines normalized to control levels (*n* = 12-31 cells per condition, **P < 0.01 and ***P < 0.001, Student's *t*-test). **(C)** Cultured hippocampal neurons (13-14 DIV) treated with control or Wnt7a for 3 hours. vGlut1 (blue) and sGluA2 (red) on dendritic spines visualized by EGFP-actin (green; scale bar: 2.5 µm). **(D)** Quantification of percentage of spines containing sGluA2 (n = 27-31 cells per condition, ***P < 0.001 Student's t-test).

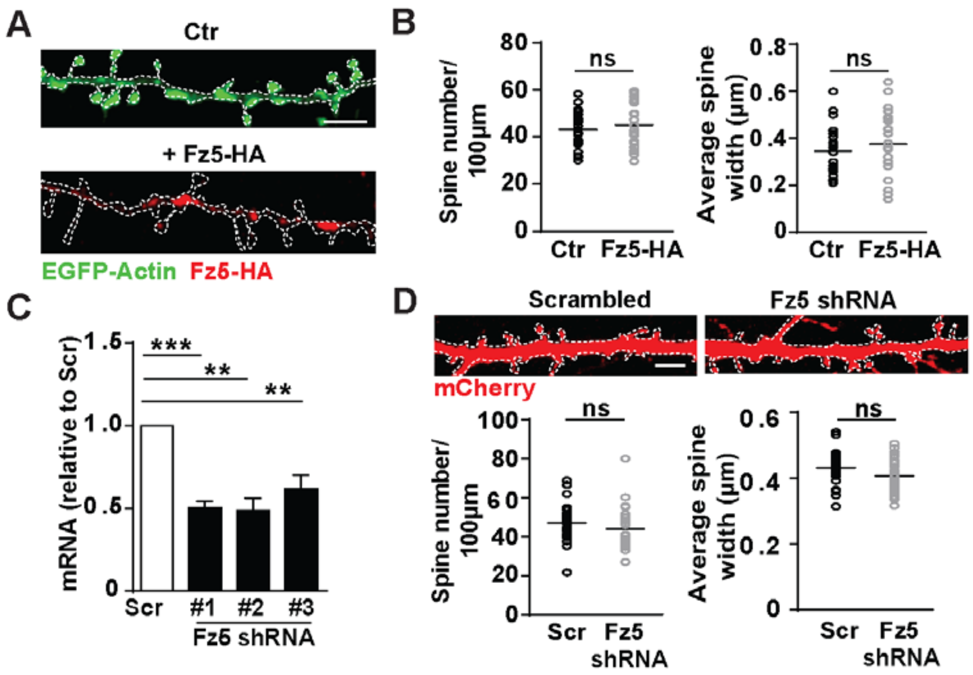

Figure S4: related to Figure 3. Fz5 receptors are not enriched at dendritic spines and do not affect spine structural plasticity. (A) Fz5-HA (red) expressing cultured hippocampal neurons (DIV13-14; scale bar: 5 µm). Note the lack of Fz5 in spines (EGFP-Actin annotated dotted lines). **(B)** Quantification of spine number and width (*n* = 28-32 cells per condition, Students *t*-test). **(C)** Fz5 mRNA levels in NRK cells transfected with scrambled or three different shRNA clones. Graph represents fold change relative to scrambled shRNA control and data expressed as mean ± SEM. Note: Fz5 shRNA clone #1 was used for functional experiments (*n* = four independent cultures, **P < 0.01, ***P < 0.001 by Students *t*-test). **(D)** Above: hippocampal neurons (13-14 DIV) expressing scrambled or Fz5 shRNAs. Neurons labelled with mCherry (scale bar: 2.5 µm). Below: quantification of spine number and width (*n* = 27 cells per condition, Students *t*-test).

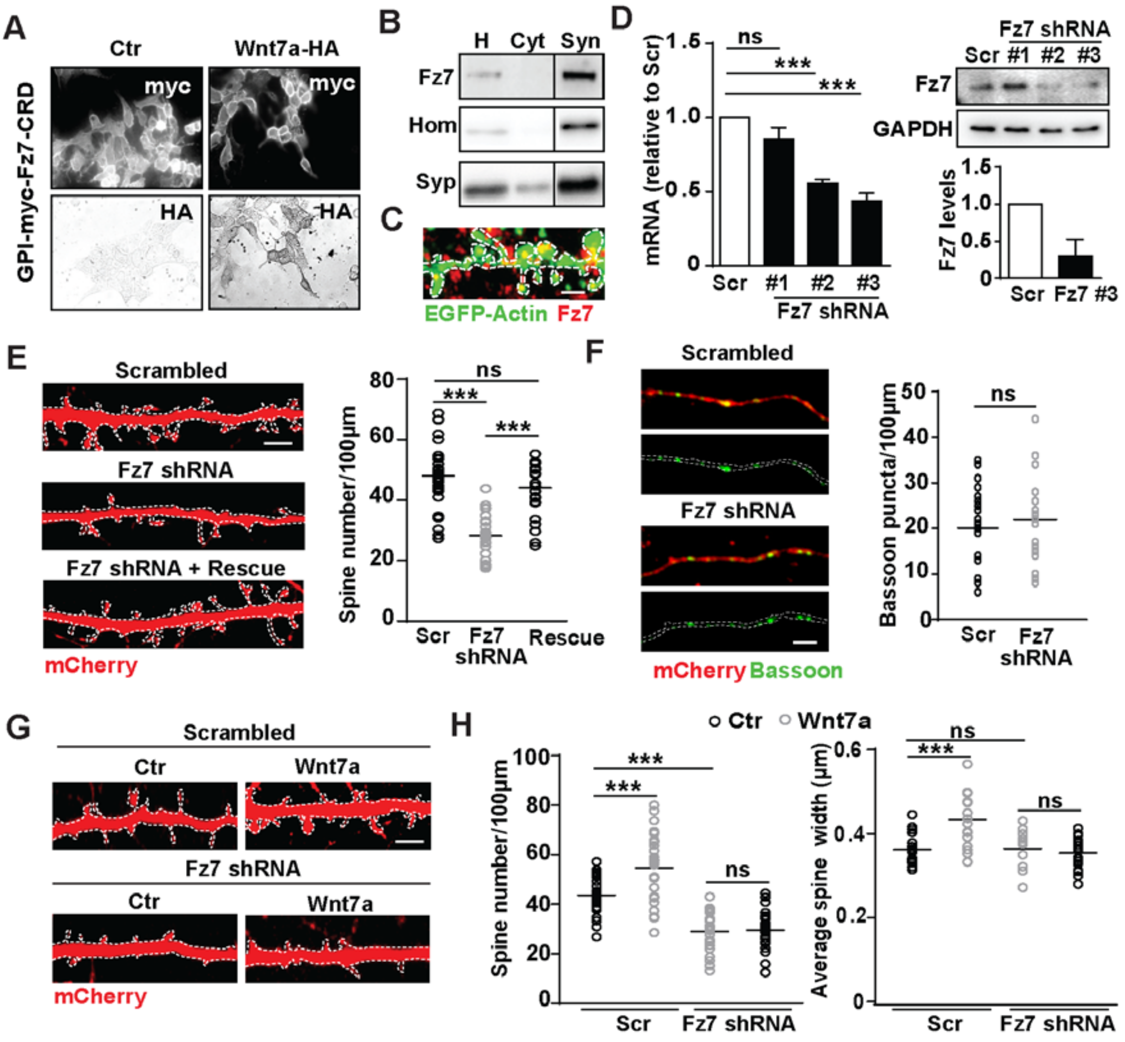

Figure S5: related to Figure 3. Fz7 receptors are enriched at spines and are required for Wnt7a-induced structural plasticity. (A) Wnt7a-HA from conditioned medium (dark staining) binds to HEK 293 cells expressing GPI-myc-Fz7-cysteine rich domain (CRD) domain. Conditioned media from EGFP expressing cells was used as control. **(B)** Representative western blot of adult rat brain homogenates (H), cytosol (Cyt) and synaptosomes (Syn) fractions showing the enrichment of Fz7, Synaptophysin (Syp; presynaptic marker) and Homer1 (postsynaptic marker) in the synaptosome fractions. **(C)** In cultured hippocampal neurons, endogenous Fz7 (red) is enriched at spines and present on the dendritic shaft. (scale bar: 1 µm). **(D)** Left: Fz7 mRNA levels (left) obtained from NRK cells transfected with scrambled control or three different shRNA clones respectively. Graphs represent fold change relative to scrambled (*n* = four independent cultures, **P < 0.01, ***P < 0.001 by Students *t*-test). Data expressed as mean ± SEM. Right: endogenous Fz7 protein levels in cultured hippocampal neurons transfected with scrambled control or three different Fz7 shRNA clones. Quantification of Fz7 shRNA clone #3, the chosen shRNA used for all studies (*n* = 2 independent cultures). Data expressed as mean ± SEM. **(E)** Left: hippocampal neurons (12-14 DIV) expressing scrambled shRNA, shRNA against Fz7 or both Fz7 shRNA and the rescue construct. Neurons labelled with mCherry (scale bar: 2.5 µm). Quantification of spine number (*n* = 27 cells per condition, ***P < 0.001 ANOVA). **(F)** Left: isolated axons of hippocampal neurons (9-11 DIV) expressing scrambled or Fz7 shRNA. Neurons labelled with mCherry and presynaptic puncta labelled with Bassoon (green, scale bar: 5 µm). Right: quantification of bassoon puncta along 100 µm of axons (*n* = 21 cells per condition, Students *t*-test) **(G)** Hippocampal neurons (12-14 DIV) expressing scrambled shRNA or shRNA against Fz7 exposed to control or Wnt7a for 3 hours. Neurons labelled with mCherry (scale bar: 2.5 µm). **(H)** Quantification of spine number (left) and size (right) in hippocampal neurons exposed to different conditions (*n* = 27 cells per condition, ***P < 0.001, ANOVA).

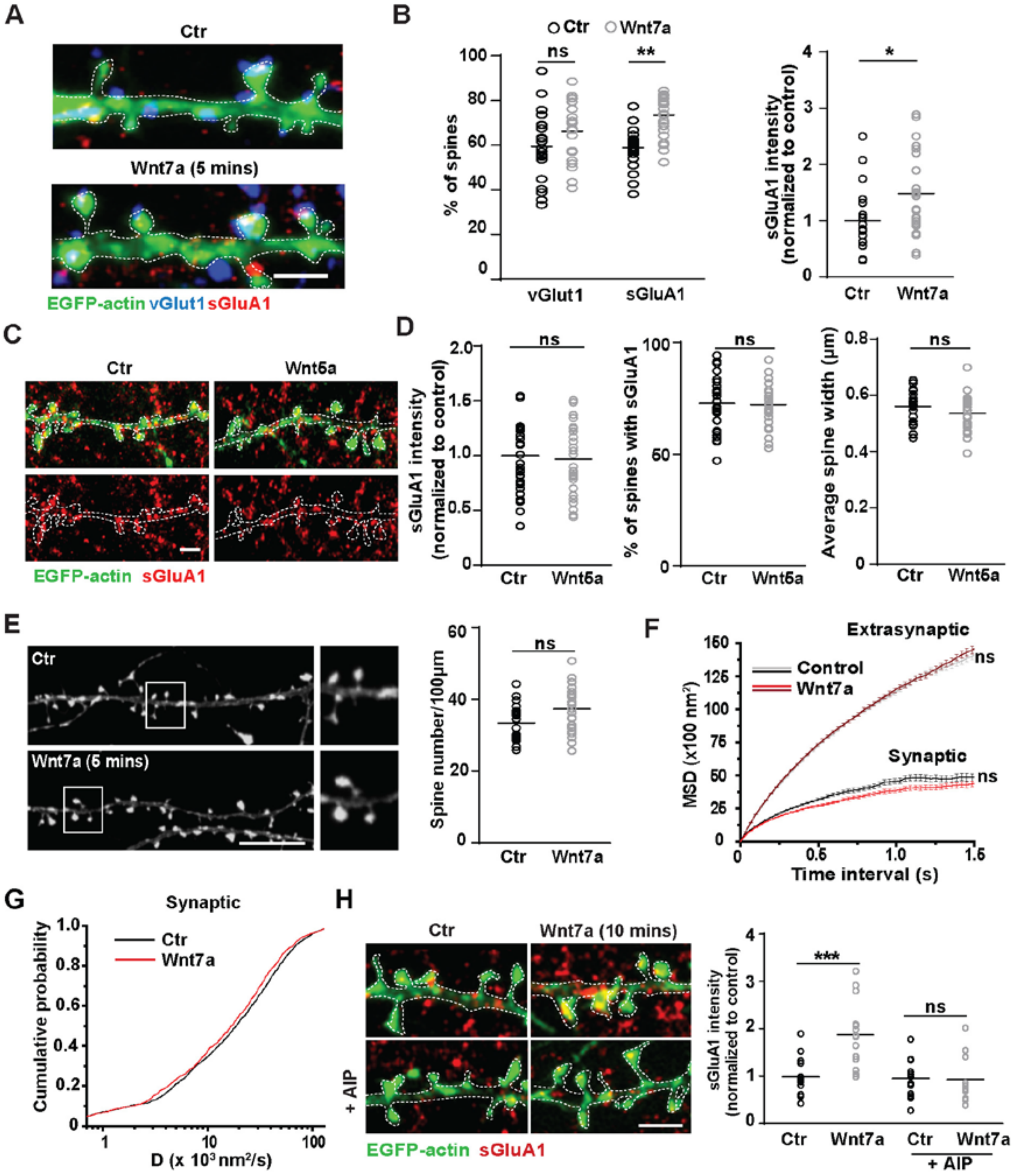

Figure S6 – related to Figures 4, 5 and 7. Wnt7a rapidly promotes the synaptic localization and diffusion of endogenous GluA1 containing AMPARs in a CaMKII dependent manner without affecting spine number. (A) Hippocampal neurons (14 DIV) treated with control or Wnt7a for 5 minutes. vGlut1 (blue) and sGluA1 (red) on dendritic spines visualized by EGFP-actin (green; scale bar: 2.5 µm). **(B)** Quantification of spines containing vGlut1 or sGluA1 puncta and the intensity of sGluA1 in spines (normalized to control) (*P < 0.05 and **P < 0.01, Students *t*-test, *n* = 23 cells per condition). **(C)** EGFP-actin–expressing hippocampal neurons (12 DIV) exposed to control or Wnt5a for 10 minutes. sGluA1 puncta labelled in red (scale bar: 2.5µm) **(D)** Quantification of sGluA1 intensity in dendritic spines (left), percentage of spines containing sGluA1 and average spine width in control or Wnt5a treated cells (*n* = 24 cells per condition, Students *t*-test). **(E)** EGFP-actin–expressing hippocampal neurons (14 DIV) exposed to control or Wnt7a for 5 minutes (left) (scale bar: 10 µm) and quantification of spine number (n = 24, Students *t*-test). **(F)** MSD plots representing the confinement of synaptic and extrasynaptic QD-GluA1 between control and Wnt7a (10 minutes). Significance measured using Students *t*-test from the area under the curve. Entire dataset *n* = 1638 (control) and 1409 (Wnt7a) of synaptic trajectories and 12350 (control) and 10459 (Wnt7a) of extrasynaptic trajectories from 4 independent experiments. **(G)** Cumulative probability of D-coefficient in log scale for QD-GluA1 at synapses in control and Wnt7a (10 minutes) treated cells. **(H)** EGFP-actin–expressing hippocampal neurons (12-14 DIV) exposed to control or Wnt7a for 10 minutes with or without the CaMKII inhibitor AIP. sGluA1 puncta labelled in red (scale bar: 2.5 µm). Quantification of sGluA1 intensity on spines (normalized to control) (n = 15 cells per condition, ***P < 0.001, ANOVA).

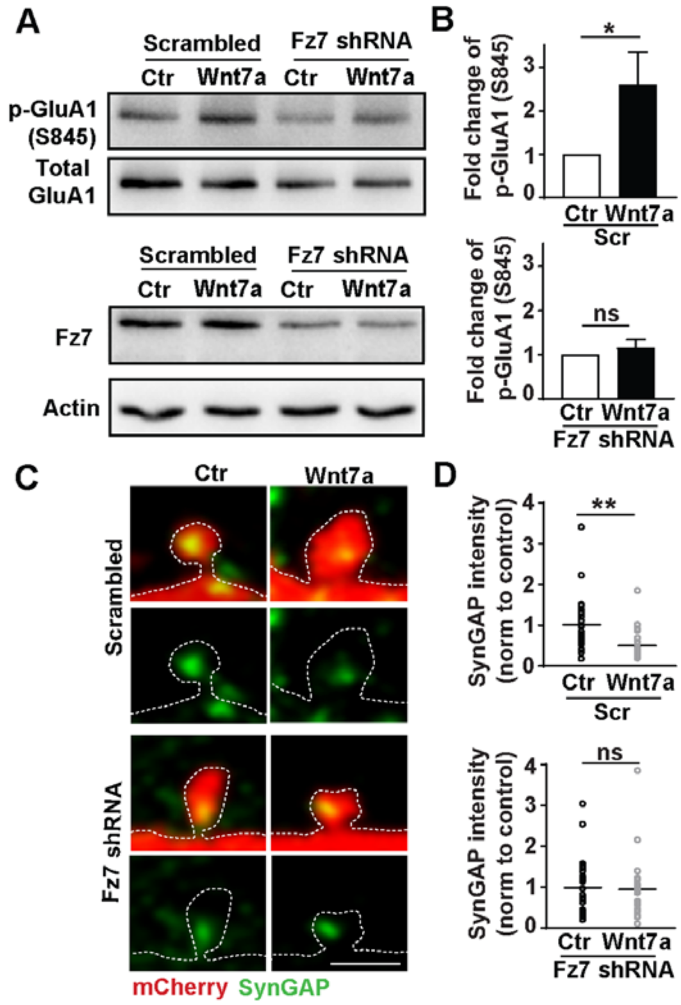

Figure S7 – related to Figures 3, 6 and 7. Fz7 receptors are required for Wnt7a-induced phosphorylation of GluA1 (S845) and SynGAP removal from dendritic spines. (A) Top: phosphorylation of GluA1 at S845 (p-GluA1) in cells infected with AAV1 scrambled or Fz7shRNA followed by 20 minutes exposure to control or Wnt7a (12 DIV). Bottom: Fz7 protein levels in cells infected with AAV1 scrambled or Fz7shRNA. **(B)** Quantification of p-GluA1 levels normalized to total GluA1 upon Wnt7a treatment. Graphs show fold changes in S845 levels relative to controls. AAV1 scrambled (above) and Fz7 shRNA (below) infected cells. (*P < 0.05, Student's t-test, *n* = 5 experiments per data set). Data expressed as mean ± SEM. **(C)** Evaluation of endogenous SynGAP (green) in spines from neurons expressing mCherry scrambled or Fz7 shRNA (12 DIV) and exposed to control or Wnt7a for 10 minutes (scale bar: 1 µm). **(D)** Quantification of SynGAP intensity within small to medium spines normalized to control levels in scrambled (top) or Fz7 shRNA (bottom) transfected cells (**P < 0.01 by Students *t*-test, *n* = 25 cells per condition).

SUPPLEMENTAL EXPERIMENTAL PROCEDURES

Cultures and Constructs

Primary hippocampal cultures were prepared from E18 embryos of Sprague-Dawley rats as described previously (Dotti et al, 1988) and maintained in Neurobasal medium supplemented with N-2 (Life Technologies, Paisley, UK), B27 (Life Technologies), D-glucose and L-glutamine for 13-14 days in vitro (DIV).

Scrambled (5'-GGCGTTACGTCCTAACATGCG-3'), Fz5 shRNA (5'-GAACTCGCTACGAGGCTTTGT-3') and Fz7 shRNA (5'-GGTGGGTCATTCTTTCCCTCA-3') target sequences were cloned into an AAV vector expressing mCherry. KD of Fz7 will only affect those neurons where the shRNA is expressed (a cell autonomous phenotype) therefore, we carefully performed our structural and functional studies on cells specifically labelled with mCherry.

Organotypic slices were maintained for 2 weeks in MEM based culture media. Fz7 functional studies were performed using the same scrambled and shRNA target sequences as above and packaged with AAV1. Infection of brain slices was performed one day after culturing using a pressure micro injector (PMI-100, Dagan Corporation, Minneapolis, USA).

Drug Treatment

Purified recombinant Wnt7a (100-150 ng/mL; PeproTech, London, UK) was applied to neurons in Neurobasal medium at 37ºC for different time periods depending on the experiment. Bovine serum albumin (BSA) was used as a control. For Protein Kinase A (PKA) inhibition, neurons were pre-incubated with myristoylated PKI (14-22) amide (5 µM; Tocris, Bristol, UK) for 15 minutes prior to and during Wnt7a treatment. For CaMKII inhibition, neurons were treated with myristoylated AIP (1 µM; Calbiochem, Darmstadt, Germany) during Wnt7a treatment.

Real-time PCR

Total RNA from NRK cells was extracted with Trizol Reagent (Life Technologies) and Direct-zol™ (Zymo Research, California, USA). Briefly, cells were washed with cold PBS and collected with Trizol. Once homogenized, 1 volume of ethanol absolute was added to each sample and loaded into Direct-zol columns. RNA was extracted following the manufacturer's instructions, including in column DNases treatment. RNA was quantified by absorbance at 260 nm using a Nanodrop ND-100 (Thermo Scientific, Dartford, UK). Retrotranscription to first strand cDNA was performed using RevertAid H Minus First Strand cDNA Synthesis kit (Thermo Scientific). 5 ng of synthesized cDNA was used to perform the qPCR using GoTaq® qPCR Master Mix (Promega, Wisconsin, USA) in a CFX96 Touch™ Real-Time PCR Detection System (Bio-Rad Laboratories, Inc.). Each sample was run in triplicates.

To detect Fzd7 and Fzd5 transcripts we used the primers at 0,5 µM final concentration (primers purchased from Sigma-Aldrich, Dorset, UK: Fzd7 Fw 5'- GCAGTGGCTGAAAAGACTCC- 3'; Fzd7 Rv 5'-CAGTTAGCATCGTCCTGCAA-3'; Fzd5 Fw 5'- TCTGTTATGTGGGCAACCAA-3'; Fzd5 Rv 5'-CCAAGACAAAGCCTCGTAGC-3'). Gapdh was used as a housekeeping gene (Sigma-Aldrich; Gapdh Fw 5'-ATGGCCTTCCGTGTTCCTAC-3'; Gapdh Rv 5'-CATACTTGGCAGGTTTCTCCA-3').

Chemical Long-Term Potentiation

Long-term potentiation (LTP) was induced in cultured hippocampal neurons with a glycine-mediated form of chemical LTP (cLTP) as previously described (Fortin et al, 2010, Stamatakou et al, 2013). Prior to stimulation, neurons were incubated at room temperature for 20-30 minutes in control solution (125 mM NaCl, 2.5 mM KCl, 1 mM $MgCl_2$, 2 mM $CaCl_2$, 33 mM D-glucose, 5 mM HEPES, 20 µM D-APV, 3 µM strychnine, 20 µM bicuculline and 0.5 µM TTX; pH 7.4). cLTP was induced for 10 minutes at room temperature with the same solution as above but containing glycine (200 µM) without Mg^{2+}, TTX, and D-APV. After cLTP induction, neurons were returned to control solution for 5 or 60 minutes before fixation and 20 minutes before AMPAR-mediated miniature EPSC (mEPSC) recordings.

Immunofluorescence, Image Acquisition and Analyses

Hippocampal neurons were fixed with 4% paraformaldehyde (PFA)/4% sucrose in PBS for 20 minutes at room temperature, permeabilised with 0.05% Triton, blocked with 5% BSA then incubated with primary antibodies overnight at 4ºC. Primary antibodies against GFP (Cat no: 06-896, Millipore, Darmstadt, Germany), vGlut1 (Cat

no: ab5905 Millipore, Darmstadt, Germany), Wnt7a/b (Cat no: AF3460, R&D Systems, Minneapolis, Minnesota, USA), Wnt5a (Cat no: ab174963, Abcam, Cambridge, UK), mCherry (Cat no: ab167453, Abcam), Fz7 (Cat no: 06-1063 Millipore), SynGAP (Cat no: ab3344; Cat no: ab223251, Abcam), and HA tag (Cat no: 11867423001, Sigma-Aldrich) were used. For surface GluA1 (Cat no: PC246, Millipore) and GluA2 (Cat no: MAB 397, Millipore) staining, live cells were incubated with each antibody for 10-15 minutes at 37ºC before fixation. Secondary antibodies Alexa-488, Alexa-568 and Alexa-647 were from Molecular Probes, Paisley, UK. Fluorescence images of pyramidal neurons were captured by confocal microscopy. We used an Olympus FV1000 inverted confocal microscope using a 60x oil-immersion objective (NA = 1.40) producing image stacks of 162.7 X 162.7 µm or a Leica TCS SP1 confocal microscope using a 63x oil objective (NA = 1.32) and producing images stacks of 157.8 x 157.8 µm. The average z-depth of each image was ~3µm. Image acquisition was completed on the same microscope per dataset.

For each experiment, 8-12 images of cells were taken per condition and analyzed using Volocity software (Improvision, Llantrisant, UK). Dendritic spine morphology was measured manually such that for every EGFP-actin or mCherry-expressing cell, 3-4 dendrites (~50-100 µm in length each) containing roughly 100 spines were cropped from maximum projections. Spine width was quantified by placing the line tool over the maximum spine head width and the number of spines was counted and normalized to the length of the dendrite. The presence of synaptic puncta (GluA1, GluA2, SynGAP and vGlut1) adjacent or on spines was detected using custom Volocity threshold protocols and confirmed manually through visualization of each individual focal plane. Extrasynaptic puncta were defined as puncta not opposed to vGlut1 along the dendrite. Fluorescent intensity measurements were calculated on cropped spines and dendrites using custom Volocity threshold protocols and normalized to controls. SynGAP intensity was specifically measured on spines with a volume of 1.5 μm^3 or less to eliminate large spines as Wnt7a activates CaMKII in small and medium size spines but not in large ones (Ciani et al, 2011). Wnt7a/b intensity was normalized to the volume of the dendritic spine based on EGFP-Actin or mCherry labelling.

Acute hippocampal slices (400µm thick from P28 rats) were fixed in 4% PFA/4% sucrose in PBS for one hour and incubated in blocking solution (10% donkey serum, 0.02% Triton X-100 in PBS) for ~4-6 hours at room temperature. Primary antibodies against Wnt7a/b (R&D Systems) and MAP2 (Abcam) were applied overnight at 4ºC. Slices were subsequently washed in PBS and incubated with secondary antibodies Alexa-488, Alexa-568 and Alexa-647 for 2-3 hours at room temperature. To label cell nuclei, slices were incubated with Hoechst stain for 5 minutes before being mounted in Fluoromount-G (SouthernBiotech, Cambridge, UK).

Fluorescence images of the hippocampus (CA1 pyramidal cell layer and stratum radiatum) were captured on an Olympus FV1000 inverted confocal microscope using a 40x objective (NA = 0.95) and image stacks acquired with a z-step of 1 µm. Three z-stacks were taken in each hippocampal region per slice, from 3 slices per animal. Wnt7a/b and MAP2 staining was detected and fluorescence intensity calculated using custom Volocity software threshold protocols. MAP2 intensities were used to normalize Wnt7a/b signal intensity. The intensity in the stratum radiatum was measured after removing the cell bodies from the analyses.

Western Blot Analysis

Equal amounts of protein (Lowry assay) were loaded on a 10% SDS/PAGE and western blots probed with primary antibodies against total GluA1 (Cat no: 13185S Cell Signalling, Danvers, Massachusetts, USA), phospho-Ser845 (Cat no: PPS008, R&D Systems), total ERK (ERK-1&2, Cat no: M6670, Sigma-Aldrich), phospho-ERK (diphosphorylated ERK-1&2, Cat no: M8159 Sigma-Aldrich), Synaptophysin (Cat no: MAB5258, Millipore), Homer1 (Cat no: 160003, Synaptic Systems, Göttingen, Germany), Fz7 (Cat no: ab64636, Abcam), β-Actin (Cat no: 8457 Cell Signalling), GAPDH (Cat no: ab181602 Abcam), α-Tubulin (Cat no: T9026, Sigma-Aldrich). Band intensity was measured using the Gel Analysis method in ImageJ software. The level of GluA1 and ERK phosphorylation was determined by calculating the ratio of phosphorylated protein over the total protein. Phospho-ERK levels were first normalized for loading using α-Tubulin and then to total Erk levels from samples run in a separate gel in which α-Tubulin was also used as control.

Synaptosomal Preparation

Synaptosomes were prepared as previously described (Cohen et al, 1977). All steps were performed at 4°C and all buffers contained protease inhibitors. Brains from P20 rats were homogenized in Syn-PER buffer (Thermo Scientific) and centrifuged at 1,200g for 10 min. The supernatant was centrifuged at 15,000g for 20 minutes, and the pellet was resuspended in Syn-PER buffer. This sample was then layered on top of a discontinuous sucrose gradient (0.85/1.0/1.2 M sucrose in 4 mM Hepes at pH 7.4) and centrifuged at 53,000g for 2 hours in a swinging bucket (Beckman SW28). The synaptosomal fraction was taken from the 1–1.2 M sucrose interface, incubated

with an equal volume of Triton buffer (80mM Tris pH 8.0/1% Triton) for 25 minutes and then centrifuged at 21,000g for 20 minutes. The supernatant was removed and the pellet resuspended in Syn-PER buffer.

Binding Assay

HEK 293 cells were transfected with the CRD domain of human Fz7 containing a glycosyl-phosphatidylinositol (GPI) sequence (GPI-myc-Fz7-CRD). Subsequently, cells were then incubated for 1 hour at RT with control or Wnt7a-HA-containing conditioned media (from QT6 cells) then fixed with 4% PFA. Primary antibodies to HA (Sigma-Aldrich) and Myc (Sigma-Aldrich) was used followed by incubation with HRP-biotinylated (Amersham) and Alexa-488 conjugated secondary antibodies.

Electrophysiology

AMPAR-mediated mEPSCs were recorded from hippocampal neurons (250 cells/mm^2) on an upright microscope and continuously perfused at room temperature with oxygenated (95% O2/5% CO2) recording artificial cerebrospinal fluid (ACSF) solution containing (in mM): NaCl (125), $NaHCO_3$ (25), KCl (2.5), $NaHPO_4$ (1.25), D-glucose (25), supplemented with $CaCl_2$ (1), $MgCl_2$ (1), bicuculline (0.01), APV (0.05) and TTX (0.0001). Spontaneous EPSC (sEPSC) were recorded from hippocampal neurons perfused in a solution containing (in mM): NaCl (145), KCl (3), $CaCl_2$ (1.5), $MgCl_2$ (1), HEPES (10), D-glucose (10) and glycine (0.01) adjusted to pH 7.4. All cells were voltage-clamped at -60 mV in the whole cell configuration using borosilicate glass patch electrodes (5–8 MΩ) filled with an intracellular solution containing (in mM): D-gluconic acid lactone (139), HEPES (10), EGTA (10), NaCl (10), $CaCl_2$ (0.5), $MgCl_2$ (1), ATP (1) and GTP (1) adjusted to pH 7.2 with CsOH.

NMDA/AMPAR ratio experiments and input-output experiments were performed in acute transverse hippocampal slices (300 µm) from P28 rats in recording ACSF supplemented with (in mM): $CaCl_2$ (2), $MgCl_2$ (1). NMDAR EPSCs were recorded at +40 mV in containing CNQX (10µM) and bicuculline (10µM) and AMPAR EPSCs were recorded at -40 mV in the presence of bicuculline. Recording glass microelectrodes (filled with the same intracellular solution as above including 5mM QX-314) were positioned in CA1 pyramidal cell layer whilst concentric bipolar stimulating electrodes were placed in SC afferent fibres. The peak averaged NMDAR EPSCs were divided by the averaged AMPAR EPSCs.

Field EPSP (fEPSP) recordings were performed in acutely prepared transverse hippocampal slices (400µm) from P28 rats. After a 1 hour equilibration period, slices were placed into an interface-like chamber and perfused at 32ºC with oxygenated recording solution supplemented with (in mM): $CaCl^2$ (2), $MgCl^2$ (1) and DPCPX (0.01; adenosine A1 receptor blocker). Recording glass microelectrodes (resistance 1-2 MΩ) were filled with the same recording ACSF and positioned in the stratum radiatum of the CA1 region whilst concentric bipolar stimulating electrodes were placed in SC afferent fibres to record fEPSPs. Paired pulse stimuli were given 50 ms apart every 10 seconds with a stimulation strength set to approximately 50% of the strength giving a maximal response. Following a stable 20 minute baseline recording (<10% change in fEPSP slope), LTP was induced by high frequency stimulation (HFS; a single train of 100 stimuli at 100 Hz) and fEPSPs monitored for 1 hour. To block Wnts, Sfrp3 (250 ng/mL) was applied 10 minutes before HFS and present during LTP.

Pairing LTP experiments were performed in organotypic hippocampal slices (DIV 9-16) infected with AAV1-expressing scrambled or Fz7 shRNA constructs. Slices were continuously perfused at room temperature with oxygenated (95% O2/5% CO2) recording solution supplemented with (in mM): $CaCl_2$ (4), $MgCl_2$ (4), 2-chloroadenosine (2-3) and picrotoxin (0.05). Patch recording pipettes (3–8 MΩ) were filled with 140 mM cesium methanesulfonate, 8 mM CsCl, 10 mM HEPES, 2 mM Mg_2ATP, 0.3 mM Na_3GTP, 7 mM sodium phosphocreatine, spermine 0.1mM and 0.25 mM EGTA, pH 7.25. Synaptic responses were evoked with concentric bipolar stimulating electrodes placed in SC afferent fibres (single voltage pulses 200 µs). Pairing LTP was induced in CA1 neurons by pairing 3 Hz presynaptic stimulation of the Schaffer collaterals with 0 mV postsynaptic depolarization for 1.5 minutes.

All currents were recorded using an Axopatch 200B amplifier, filtered (1 kHz) and digitised (10 kHz). Data was monitored online and analyzed offline using WinEDR and WinWCP software (available free online at http://spider.science.strath.ac.uk/sipbs/software_ses.htm).

Live imaging of SEP-GluA1 and analyses

Time-lapse experiments of SEP-GluA1 were performed on an Olympus FV1000 inverted confocal microscope using a 60x oil-immersion objective (NA = 1.40) with temperature controlled chamber maintained at 37ºC.

Neurons were placed into a chamber with HEPES buffered solution containing (in mM): NaCl (120), HEPES (10), Glucose (10), $CaCl_2$ (2) and $MgCl_2$ (1) adjusted to pH 7.3 with KOH. Transfected neurons with SEP-GluA1 and mRFP (to detect spines) were identified under epifluorescence and images acquired sequentially using a maximum 10% of laser power (488 nm and 559 nm) in a 512 x 512 format, 500 Hz speed, 3-line averaging, and 2.0 optical zoom. Z-stacks (~10 frames) were acquired every minute for at least 20 minutes. Control or Wnt7a (150 ng/mL) was applied directly to neurons in HEPES buffered solution before image acquisition. Data was analyzed on the projected z-stack using ImageJ software from manually selected regions of interest (ROI) around each spine head or just below the spine on the dendritic shaft (extrasynaptic). Integrated density values (which considers the area analyzed and the fluorescence intensity) within each ROI were acquired and subtracted from background levels at each minute in the time-lapse recording. Data for each ROI were normalized to baseline values of that same ROI obtained before addition of Control or Wnt7a. Spine volume was measured as the integrated density of mRFP on a given spine normalized to the integrated density of the adjacent dendrite, to correct for any bleaching that may have occurred during live imaging.

Single-Particle Imaging of AMPARs

Hippocampal neurons were quickly rinsed and incubated with the primary antibody against GluA1 (Cat no: PC246, Millipore) for 2 minutes. Subsequently neurons were washed for 30 seconds, incubated with a biotinylated anti-rabbit IgG secondary antibody (Vector Laboratories, Peterborough, UK) for 1 minute then washed for 30 seconds. Lastly, streptavidin-coated Quantum Dots (QDs) emitting at 655 nm (QD655; 50 pM; Life Technologies) dissolved in 2% BSA in Borate buffer was applied to neurons and washed for 1 minute respectively. All incubations and washes were performed at 37ºC in Krebs solution containing (in mM): NaCl (140), KCl (4.7), MgO_2*$6H_2O$ (12), $CaCl_2$ (2.52), Glucose (11) and HEPES (5) adjusted to pH 7.4.

QD655-tagged AMPARs were imaged immediately using an Olympus IX71 inverted microscope, with a 60x objective (NA = 1.35) and halogen lamp illumination (PhotoFluor-II Metal Halide illumination system). Images were acquired using a back-illuminated electron-multiplying charge coupled device (EMCCD) camera (iXon3 885; Andor Technology, Belfast, UK) with minimum exposure (typically 30-100 ms) in 16 bits using Cairn-Metamorph Meta Imaging software (Molecular Devices, Wokingham, UK). At the start of time-lapse recordings, an image of the EGFP-actin labelled dendrite was acquired followed by an image sequence of 300 frames at 33 Hz for QD655.

Single-Particle Tracking and Quantitative Analyses of Lateral Diffusion

Single-particle tracking of QD655-tagged AMPARs involved the detection of the centre of the QD spot fluorescence by a two-dimensional Gaussian fit with a spatial resolution of 10 – 20 nm for each image frame using a Matlab (MathWorks) based software, SPTrack (Hannan et al, 2016). The Gaussian peaks of consecutive frames were joined using estimated diffusion coefficient and maximum likelihood. The mean square displacement (MSD; surface explored) of each QD was calculated using the following equation:

$$\mathrm{MSD}\,(ndt) = (N - n)^{-1}\Sigma_{\,i\,=\,1}^{\;N\,-\,n}((x_{i\,+\,n} - x_i\,)^2 + ((y_{i\,+\,n} - y_i\,)^2)$$

where x_i and y_i are the spatial co-ordinates of a QD on any image frame i, N is the total number of points in the trajectory, dt is the time interval between two successive frames (33 ms), and ndt is the time interval over which the displacement is averaged. From the MSD plot, the diffusion coefficient (a measure of how far and fast the QDs are moving), D, *for a QD* was calculated by fitting the first two to five points of the MSD plot against time with the following equation:

$$\mathrm{MSD}(t) = 4D_{\,2\text{–}5}\;t + 4\sigma_x^{\;2}$$

where σ_x is the QD localization accuracy in one direction.

Synaptic regions were visualized in EGFP-actin-expressing neurons. Images were processed and thresholded to observe spines using ImageJ plug-in (TopHatFilter). QD trajectories that co-localized with spines were defined as synaptic. QD data was analyzed using Origin (Version 6) and built-in functions in Matlab. Immobile QDs were defined as having a D-coefficient less than 0.007 $\mu m^2/s$ as previously described (Tardin et al, 2003).

SUPPLEMENTAL REFERENCES

Tardin, C, Cognet, L, Bats, C, Lounis, B, and Choquet, D. (2003). Direct imaging of lateral movements of AMPA receptors inside synapses. EMBO J *22*, 4656-4665.
